# Exome sequencing reveals predominantly de novo variants in disorders with intellectual disability (ID) in the founder population of Finland

**DOI:** 10.1007/s00439-021-02268-1

**Published:** 2021-03-12

**Authors:** Irma Järvelä, Tuomo Määttä, Anushree Acharya, Juha Leppälä, Shalini N. Jhangiani, Maria Arvio, Auli Siren, Minna Kankuri-Tammilehto, Hannaleena Kokkonen, Maarit Palomäki, Teppo Varilo, Mary Fang, Trevor D. Hadley, Angad Jolly, Tarja Linnankivi, Ritva Paetau, Anni Saarela, Reetta Kälviäinen, Jan Olme, Liz M. Nouel-Saied, Diana M. Cornejo-Sanchez, Lorida Llaci, James R. Lupski, Jennifer E. Posey, Suzanne M. Leal, Isabelle Schrauwen

**Affiliations:** 1 Department of Medical Genetics, University of Helsinki, P.O. Box 720, 00251 Helsinki, Finland; 2 Disability Services, Joint Authority for Kainuu, Kajaani, Finland; 3 Center for Statistical Genetics, Sergievsky Center, Taub Institute for Alzheimer’s Disease and the Aging Brain, and the Department of Neurology, Columbia University Medical Center, New York, NY USA; 4 Eskoo The Center for Disability Empowerment, Seinäjoki, Finland; 5 Human Genome Sequencing Center, Baylor College of Medicine, Houston, TX 77030 USA; 6 KTO-Special Welfare District of Varsinais-Suomi, Paimio, Finland; 7 Päijät-Häme Joint Municipal Authority, Lahti, Finland; 8 PEDEGO, University of Oulu, Oulu, Finland; 9 Department of Clinical Genetics, Turku University Hospital, Turku, Finland; 10 Kanta-Häme Central Hospital, Hämeenlinna, Finland; 11 Department of Clinical Genetics, University of Turku, Turku, Finland; 12 Northern Finland Laboratory Centre NordLab and Medical Research Centre, Oulu University Hospital and University of Oulu, Oulu, Finland; 13 Department of Radiology, Helsinki University Hospital, Helsinki, Finland; 14 Baylor College of Medicine, Houston, TX 77030 USA; 15 Department of Molecular and Human Genetics, Baylor College of Medicine, Houston, TX 77030 USA; 16 Department of Child Neurology, University of Helsinki and Helsinki University Hospital, Helsinki, Finland; 17 Institute of Clinical Medicine, School of Medicine, Faculty of Health Sciences, University of Eastern Finland, Kuopio, Finland; 18 Kuopio Epilepsy Center, Kuopio University Hospital, Neurocenter, Finland; 19 Department of Child Neurology, Vaasa Central Hospital, Vaasa, Finland; 20 Division of Biology and Biomedical Sciences, Washington University in St. Louis, St. Louis, MO 63110 USA; 21 Department of Pediatrics, Baylor College of Medicine, Houston, TX 77030 USA; 22 Texas Children’s Hospital, Houston, TX 77030 USA

## Abstract

The genetics of autosomal recessive intellectual disability (ARID) has mainly been studied in consanguineous families, however, founder populations may also be of interest to study intellectual disability (ID) and the contribution of ARID. Here, we used a genotype-driven approach to study the genetic landscape of ID in the founder population of Finland. A total of 39 families with syndromic and non-syndromic ID were analyzed using exome sequencing, which revealed a variant in a known ID gene in 27 families. Notably, 75% of these variants in known ID genes were de novo or suspected de novo (64% autosomal dominant; 11% X-linked) and 25% were inherited (14% autosomal recessive; 7% X-linked; and 4% autosomal dominant). A dual molecular diagnosis was suggested in two families (5%). Via additional analysis and molecular testing, we identified three cases with an abnormal molecular karyotype, including chr21q22.12q22.2 uniparental disomy with a mosaic interstitial 2.7 Mb deletion covering *DYRK1A* and *KCNJ6.* Overall, a pathogenic or likely pathogenic variant was identified in 64% (25/39) of the families. Last, we report an alternate inheritance model for 3 known ID genes (*UBA7*, *DDX47*, *DHX58*) and discuss potential candidate genes for ID, including *SYPL1* and *ERGIC3* with homozygous founder variants and de novo variants in *POLR2F* and *DNAH3.* In summary, similar to other European populations, de novo variants were the most common variants underlying ID in the studied Finnish population, with limited contribution of ARID to ID etiology, though mainly driven by founder and potential founder variation in the latter case.

**Supplementary Information:**

The online version contains supplementary material available at 10.1007/s00439-021-02268-1.

## Introduction

It is estimated that variants that affect the functions of more than 2500 genes can give rise to ID, and roughly half of these genes remain unknown. Identifying the genetic etiology of ID has been complicated by extreme genetic heterogeneity. In most studies from mixed populations, d*e novo* variants have been reported to be the most common cause of ID (Rauch et al. 2012; de Ligt et al. 2012) whereas X-chromosomal ID (XLID) contributes 10–12% of cases (de Brouwer et al. 2007). Most evidence for ARID genes has been obtained from populations where consanguineous marriages are common (Monies et al. 2017; Martin et al. 2018) whereas data about genetic variants underlying ARID are rare in outbred populations (Martin et al. 2018).

Founder populations can serve as a middle ground between mixed and consanguineous populations in the identification of ARID genes where the enrichment of a disease allele is strongly affected by genetic drift, and founder effects. The Finnish population represents a founder population where nearly 40 rare autosomal recessive (AR) diseases with one founder variant have enriched (Peltonen et al. 1999). To further dissect the landscape of the genetic causes underlying ID in a founder population, a genomic sequence-based approach of exome sequencing (ES) was used.

## Methods

A total of 39 families with mild to profound ID, and both non-syndromic and syndromic forms, were enrolled in the study. Of them 27 were trios and 12 had one parent and/or one sibling available for the analysis. Affected individuals were clinically evaluated by a child neurologist and clinical geneticist for the study. Photographs display syndromic features from affected individuals and in relevant cases, MRI was also obtained (Figure S1& S2). The parents or legal guardians of all patients and their healthy siblings in this study provided written informed consent to participate and to publish photos of the patients. The study was approved by the ethics committees of the Hospital District of Helsinki and Uusimaa and the Institutional Review Boards of Columbia University (IRB-AAAS3433) and Baylor College of Medicine (protocol H-29697).

### Exome sequencing (ES) and bioinformatic analysis

Exomic libraries were prepared using either the SureSelect Human All Exon V6 kit (Agilent Technologies, Santa Clara, CA, USA), the TruSeq DNA exome kit (Illumina Inc, San Diego, CA, USA) or the Baylor College of Medicine Human Genome Sequencing Center VCRome 2.1 design (42 Mb Nimblegen, Cat. No. 06266380001). 100 bp paired-end sequencing was performed on a HiSeq2500/4000/2000 instrument (Illumina Inc, San Diego, CA, USA). Details on bioinformatic analyses for both single nucleotide variants (SNVs), small insertion/deletions (InDels), copy number variants (CNVs) and variant filtering can be found in the supplementary methods (Supplemental Methods). In short, rare variants following several inheritance models (e.g. AR, Autosomal Dominant, X-linked) with a predicted effect on protein function or pre-mRNA splicing were retained. Known and candidate genes for ID were prioritized (sysID database; https://sysid.cmbi.umcn.nl/), and if no known or candidate genes were found, variants were assessed further using additional annotations such as gene expression and literature. Sanger sequencing was performed using an ABI3130XL Genetic Analyzer to verify candidate SNV and InDel variants and to examine segregation amongst the family members that were not exome sequenced. The classification of variants is based on the American College of Medical Genetics and Genomics (ACMG) recommendations (Richards et al. 2015).

### Molecular karyotyping

Molecular karyotyping was performed in FIN10. In short, microarray analysis was performed from DNA extracted from a lymphoblastoid cell line using the HumanCytoSNP-12 (v2.1) (Illumina, San Diego, CA). SNP genotype analysis of FIN10 and her parents’ samples were done to evaluate the origin of deletion and uniparental disomies. FISH-analysis was done both from uncultured (*n* = 200 interphases) and cultured (*n* = 300 interphases, 25 metaphases) peripheral blood lymphocytes using a probe mix detecting DNA-sequences from the *DYRK1A* gene region and from the 21q21.1 control region. Additional details are available in the Supplementary information.

### Runs of homozygosity analysis

Runs of homozygosity and inbreeding were assessed using plink(v1.90)(Chang et al. 2015) in the probands of the Finnish families, and an in-house collection of unrelated samples of European (*N* = 15; outbred) and South Asian ethnicity (*N* = 133; inbred) exome sequenced with the SureSelect Human All Exon V6 kit. In short, InDels were removed and only SNVs with a 90% genotyping rate, Hardy–Weinberg Equilibrium *p* value > 0.00001 and MAF > 0.01 were retained. One Mb or larger runs of homozygosity were assessed across the genome using a sliding window (5 Mb; 50 SNVs). Inbreeding coefficients were calculated for each sample using three different methods (Fhat 1–3) after additional filtering (MAF > 0.05) and linkage disequilibrium pruning (window size: 100; step size 10; *r*^2^ > 0.5)(Chang et al. 2015). A Kruskal–Wallis test was done to compare > 2 groups. Post-hoc analysis of pairwise comparisons was done with the Wilcoxon rank sum test with multiple testing adjustments (false discovery rate). A *T* test or Mann–Whitney *U* test was used to compare 2 groups.

## Results

Detailed phenotypic and clinical characteristics of all patients are provided in the Supplementary information. Following the analysis of the ES data, we identified a total of seven previously reported pathogenic (P) variants, 11 novel pathogenic or likely pathogenic (LP) variants, and four variants of unknown significance (VUS) in known genes in 19 families with neurodevelopmental disorders (Tables 1 and 2; Suppl information; Figures S1A & S1B). Additionally, six novel variants associated with a phenotypic expansion beyond that characterized the known disease gene and three variants with an alternate inheritance model were identified (Tables 1 and 2; Figures S1C & S1D). Nine novel candidate genes for ID were also found (Table 1, 2 and 3; Table S1; Figure S1E). For three cases where standard ES analysis did not reveal a putatively causal result, CNV analysis of ES data and/or molecular karyotyping revealed a rearrangement, of 1.25 Mb del, mosaic UPD21q22.12–22, 2.7 Mb del, and 106 kb del, in chromosomes 16, 21, and 22, respectively (Table 1; Figure S1F & S3).

**Table 1:** Overview of genomic variants identified in this study

Patient ID	Gender	Age at diagnosis	Gene	Variant	Inheritance	Severity of ID or DD	Phenotype features
Known gene—known variant							
FIN3-3	M	12	*PPP2R5D*	NM_006245.4:c.592G > A:p.(E198K)	Suspected de novo*	Severe	Classical
FIN11-3	F	24	*CYFIP2*	NM_001291722.2:c.1993G > A:p.(E665K)	De novo	Moderate	Mild phenotype, no epilepsy
FIN17-3	M	18	*MED12*	NM_005120.3:c.2881C > T:p.(R961W)	X-linked	Mild	Classical
FIN24-3	F	14	*ACTB*	NM_001101.5:c.1043C > T:p.(S348L)	De novo	Mild	Classical
FIN37-3	F	12	*DYNC1H1*	NM_001376.5:c.4700G > A:p.(R1567Q)	De novo	Severe	Classical
FIN52-1	F	53	*HUWE1*	NM_031407.7:c.9208C > T:p.(R3070C)	X-linked (suspected de novo)*	Severe	Classical, female
Known gene—novel variant							
FIN6-3	F	10	*HNRNPK*	NM_031263.4:c.1294delG:p.(D432fs)	Suspected de novo*	Moderate	Classical, high pain tolerance
FIN12-2	M	28	*ARX*	NM_139058.3:c.558_560dup:p.(P187dup)	X-linked***	Moderate	Resembles Partington's disease
FIN14-3	M	35	*CTBP1*	NM_001328.3:c.158_160del:p.(F53del)	De novo	Severe	Frontal bossing, growth retardation, cortical atrophy, no enamel defect
FIN20-3	M	20	*CHAMP1*	NM_032436.4:c.1858A > T:p.(K620*)	De novo	Moderate—severe	Frontal hypoplasia in MRI
FIN33-3	M	12	*RAI1*	NM_030665.4:c.3594G > T:p.(R1198S)	De novo	Mild	Classical
FIN36-3	F	30	*LAMB1*	NM_002291.2:c.5002delG:p.(E1668fs) + c.2315-28A > G (splicing branch point)	AR (comp het)	Moderate	Cobblestone cortical malformation, cystic lesions in cerbellum in MRI
FIN38-3	F	9	*CRADD*	NM_003805.5:c.2 T > C:p.(M1?) + NM_003805.5:c.509G > A:p.(R170H)^^^	AR (comp het)	Mild	Pachygyria in MRI
FIN42-3, FIN42-7	M, M	20, 12	*P4HTM*	NM_177939.2:c.1238C > T:p.(P413L)	AR (homozygous)	Severe-profound	Increased BMI
FIN49-1	M	74	*SCN1A*	NM_001202435.3:c.1891A > G:p.(M631V)	Suspected de novo**	Profound	Crunched gait
FIN53-1	F	72	*TRIO, SON*	NM_007118.4:c.3908C > T:p.(T1303I) + NM_001291412.2:c.953A > C:p.(Q318P)	Suspected de novo**	Moderate	Dual molecular diagnosis
FIN-ID4-3	M	15	*KIAA2022*	NM_001008537.3:c.3244C > T:p.(Q1082*)	X-linked (de novo)	Severe	Gastroesofageal reflux
FIN-ID8-3	F	21	*GRIN2A*	NM_001134408.2:c.452_463del:p.(I151_A155delinsT)	AD inherited	Moderate	Dual diagnosis: classical epilepsy, unknown syndrome
FIN-ID10-3	F	22	*ANKRD11*	NM_001256183.2:c.6793delG:p.(A2265fs)	De novo	Moderate	Dual diagnosis, Mayer-Rokitansky
Known gene—novel variant—phenotypic expansion							
FIN5-3	M	39	*SAMD9L*	NM_001350084.2:c.2722A > G:p.(I908V)	De novo	Moderate	Expansion: ataxia-pancytopenia not found
FIN8-3	M	41	*BCL11A*	NM_022893.4:c.977C > A:p.(T326K)	De novo	Moderate	Expansion: fHb normal
FIN28-3	F	17	*MECP2*	NM_001110792.2:c.1174_1199del:p.(V392fs)	X-linked (suspected de novo)*	Mild	Expansion: mild phenotype
FIN35-3	F	17	*MYT1, (COL9A2)^*	NM_004535.3:c.790G > C:p.(E264Q) + NM_001852.4:c.1145G > T:p.(G382V)	De novo	Moderate	Expansion: extended phenotype, dual diagnosis
FIN-AIC3-3	F	16	*ZC3H14*	NM_207661.2:c.1177 + 9 T > C (splice region)	AR (homozygous)	Severe	Expansion: severe multisystem disease
Alternate inheritance model							
FIN7-3	M	41	*UBA7*	NM_003335.3:c.1904 + 3A > G (splicing)	Mosaic	Moderate	Novel phenotype
FIN46-3	M	50	*DDX47*	NM_016355.4:c.1129C > A:p.(R377S)	suspected de novo^^	Profound	Severe newborn disease
FIN-ID9-3	M	27	*DHX58*	NM_024119.3:c.1910G > A:p.(S637N)	De novo	Severe	Severe newborn disease
Candidate variants In potential novel genes							
FIN4-3	M	26	*NTRK1*	NM_002529.3:c.2271C > G:p.(Y757*) + c.2271C > T:p.(Y757 =) (mixture of both variants)	Mosaic	Moderate-severe	Lennox epilepsy
FIN21-3	M	31	*SYPL1*	NM_006754.4:c.152G > A:p.(C51Y)	AR (homozygous)	Mild	Novel phenotype
FIN23-3	M	20	*ITPR2*	NM_002223.4:c.541A > G:p.(K181E)	De novo	Mild-moderate	Resembles Gillespie syndrome (ITPR1)
FIN27-3	M	17	*ZKSCAN1*	NM_001287054.3:c.1302C > G:p.(F434L)	De novo	Severe	Novel syndrome
FIN32-3	F	35	*ZFR*	NM_016107.5:c.2667C > G:p.(D889E)	De novo	Mild	Novel phenotype
FIN45-3	F	51	*POLR2F*	NM_001301130.2:c.294-2A > G (splicing)	De novo	Profound	Severe newborn disease
FIN47-2	M	55	*DNAH3*	NM_017539.2:c.11965A > G:p.(I3989V)	Suspected de novo^^	Profound	Severe newborn disease
FIN-ID3-1, FIN-ID3-3	F, M	68, 56	*ERGIC3*	NM_015966.3:c.717 + 1G > A (splicing)	AR (homozygous)	Mild	Novel phenotype
FIN-AIC2-3	F	31	*KIF1B*	NM_183416.4:c.2066C > T:p.(P689L) + NM_015074.3:c.2543C > T:p.(P848L)	AR (comp het)	Severe	Severe newborn disease
Structural variants							
FIN10-3	F	23	*21qdel*	UPD21q22.12–22, 2.7 Mb del (DYRK1A; KCNJ6)	Mosaic	Severe	Resembles DYRK1 phenotype
FIN43-3	M,M	2	*16p13.11del*	1.25 Mb deletion (NDE1) paternal allele	AR (heterozygous; no second allele found)	Severe	Microcephaly, simplified gyral pattern
FIN48-2	M	62	*22q13.33del*	106 kb del (SHANK3)	Suspected de novo*	Severe	Classical

^*^Suspected de novo; Heterozygous in affected proband, however DNA of one of the unaffected parent(s) unavailable to confirm this

^**^Suspected de novo; Heterozygous in affected proband, however DNA of both unaffected parent(s) unavailable to confirm this; Variant was excluded from unaffected sibling

^***^variant also present in brother with trisomie 21

^not a known ID gene but could contribute to the skeletal phenotype in this patient

^^Suspected de novo; Heterozygous in affected proband, however DNA of one of the unaffected parent(s) unavailable to confirm this. Variant was excluded from unaffected sibling

^^^R170H is a known pathogenic variant in trans with novel variant NM_003805.5:c.2 T > C:p.(M1?)

**Table 2:** Annotations of SNV/InDel variants, including bioinformatic predictions, variant frequency and ACMG classification of variants

Patient ID	*Gene*	Variant	ACMG	Variant type	GnomAD PopMax*	GnomAD FIN*	GnomAD NFE*	CADD	GERP + + RS	ADA SCORE	HSF
Known gene—known variant											
FIN3	*PPP2R5D*	NM_006245.4:c.592G > A:p.(E198K)	P	Missense	–	–	–	33	5.84	–	–
FIN11	*CYFIP2*	NM_001291722.2:c.1993G > A:p.(E665K)	P	Missense	–	–	–	35	5.63	–	–
FIN17	*MED12*	NM_005120.3:c.2881C > T:p.(R961W)	P	Missense	–	–	–	35	5	–	–
FIN24	*ACTB*	NM_001101.5:c.1043C > T:p.(S348L)	P	Missense	–	–	–	30	4.66	–	–
FIN37	*DYNC1H1*	NM_001376.5:c.4700G > A:p.(R1567Q)	P	Missense	–	–	–	34	4.88	–	–
FIN38	*CRADD*	NM_003805.5:c.509G > A:p.(R170H)**	P	Missense	0.0049	0.0049	1.00E-04	27	4.69	–	–
FIN52	*HUWE1*	NM_031407.7:c.9208C > T:p.(R3070C)	P	Missense	–	–	–	34	5.88	–	–
Known gene—novel variant											
FIN6	*HNRNPK*	NM_031263.4:c.1294delG:p.(D432fs)	P	Frameshift indel	–	–	–	–	–	–	–
FIN12	*ARX*	NM_139058.3:c.558_560dup:p.(P187dup)	VUS	Nonframeshift indel	–	–	–	–	–	–	–
FIN14	*CTBP1*	NM_001328.3:c.158_160del:p.(F53del)	LP	Nonframeshift indel	–	–	–	–	–	–	–
FIN20	*CHAMP1*	NM_032436.4:c.1858A > T:p.(K620*)	P	Nonsense	–	– –	–	40	5.6	–	–
FIN33	*RAI1*	NM_030665.4:c.3594G > T:p.(R1198S)	LP	Missense	–	–	–	14.55	2.1	–	–
FIN36	*LAMB1*	NM_002291.2:c.5002delG:p.(E1668fs)	LP	Frameshift indel	4.62E-05	4.62E-05	0	–	–	–	–
FIN36	*LAMB1*	NM_002291.2:c.2315-28A > G	VUS	Splicing (3′ branch point)	0.0024	0.0024	1.86E-05	2.793	-4.39	–	Alteration of auxiliary sequences; New Donor splice site; possible branch point area
FIN38	*CRADD*	NM_003805.5:c.2 T > C:p.(M1?)	P	Start loss	–	–	–	25.6	5.19	–	–
FIN42	*P4HTM*	NM_177939.2:c.1238C > T:p.(P413L)	LP	Missense	0.0006	0.0006	0	34	5.53	–	–
FIN49	*SCN1A*	NM_001202435.3:c.1891A > G:p.(M631V)	VUS	Missense	–	–	–	0.853	3.88	–	–
FIN53	*TRIO*	NM_007118.4:c.3908C > T:p.(T1303I)	LP	Missense	–	–	–	32	6.03	–	–
FIN53	*SON*	NM_138927.4:c.6869A > C:p.(Q2290P)	VUS	Missense	–	–	–	23.4	5.85	–	–
FIN-ID4	*KIAA2022*	NM_001008537.3:c.3244C > T:p.(Q1082*)	P	Nonsense	–	–	–	39	5.28	–	–
FIN-ID8	*GRIN2A*	NM_001134408.2:c.452_463del:p.(I151_A155delinsT)	LP	Nonframeshift indel	–	–	–	–	–	–	–
FIN-ID10	*ANKRD11*	NM_001256183.2:c.6793delG:p.(A2265fs)	P	Frameshift	–	–	–	–	–	–	–
Known gene—novel variant—phenotypic expansion											
FIN5	*SAMD9L*	NM_001350084.2:c.2722A > G:p.(I908V)	LP	Missense	–	–	–	11.85	5.22	–	–
FIN8	*BCL11A*	NM_022893.4:c.977C > A:p.(T326K)	LP	Missense	–	–	–	26	5.84	–	–
FIN28	*MECP2*	NM_001110792.2:c.1174_1199del:p.(V392fs)	P	Frameshift indel	–	–	–	–	–	–	–
FIN35	*MYT1*	NM_004535.3:c.790G > C:p.(E264Q)	LP	Missense	–	–	–	17.3	3.77	–	–
FIN35	*COL9A2^*	NM_001852.4:c.1145G > T:p.(G382V)	LP	Missense	–	–	–	33	5.74	–	–
FIN-AIC3	*ZC3H14*	NM_207661-2:c.1177 + 9 T > C(splice region)	VUS	Splicing	0.0035	0.0035	0.0005	14.22	1.59	–	5′ donor; no effect on splicing predicted
Alternate inheritance model											
FIN7	*UBA7*	NM_003335.3:c.1904 + 3A > G (splicing)	NA (VUS)	Splicing	–	–	–	18.29	3.58	0.9332	Broken WT Donor Site
FIN46	*DDX47*	NM_016355.4:c.1129C > A:p.(R377S)	NA (VUS)	Missense	–	–	–	30	5.75	–	–
FIN-ID9	*DHX58*	NM_024119.3:c.1910G > A:p.(S637N)	NA (VUS)	Missense	1.77E-05	0	1.77E-05	11.78	3.12	–	–
Candidate variants In potential novel genes											
FIN4	*NTRK1*	NM_002529.3:c.2271C > G:p.(Y757*) and c.2271C > T:p.(Y757 =) (mixture of both variants)	NA (VUS)	Nonsense	–	–	–	38	0.887	–	–
FIN21	*SYPL1*	NM_006754.4:c.152G > A:p.(C51Y)	NA (VUS)	Missense	0.0064	0.0064	0.0007	28.8	4.97	–	–
FIN23	*ITPR2*	NM_002223.4:c.541A > G:p.(K181E)	NA (VUS)	Missense	–	–	–	28.7	5.02	–	–
FIN27	*ZKSCAN1*	NM_001287054.3:c.1302C > G:p.(F434L)	NA (VUS)	Missense	–	–	–	27.8	4.19	–	–
FIN32	*ZFR*	NM_016107.5:c.2667C > G:p.(D889E)	NA (VUS)	Missense	–	–	–	23.9	5.49	–	–
FIN45	*POLR2F*	NM_001301130.2:c.294-2A > G (splicing)	NA (VUS)	Splicing	–	–	–	23.2	4.42	0.7508	New cryptic 3′ Acceptor splice site -2
FIN47	*DNAH3*	NM_017539.2:c.11965A > G:p.(I3989V)	NA (VUS)	Missense	–	–	–	25.8	5.55	–	–
FIN-ID3	*ERGIC3*	NM_015966.3:c.717 + 1G > A (splicing)	NA (VUS)	Splicing	0.0002	0.0002	0	28.2	4.62	1	Broken WT Donor Site
FIN-AIC2	*KIF1B*	NM_183416.4:c.2066C > T:p.(P689L)	NA (VUS)	Missense	0.0007	0.0007	2.64E-05	19.56	6.13	–	–
FIN-AIC2	*KIF1B*	NM_015074.3:c.2543C > T:p.(P848L)	NA (VUS)	Missense	–	–	–	24.4	5.74	–	–

*Based on gnomADv2.1.1 exomes

**Known pathogenic variant in trans with novel variant NM_003805.5:c.2 T > C:p.(M1?)

^Not a known ID gene but could contribute to the skeletal phenotype seen in this family

**Table 3:** Gene function and literature description of novel candidate genes

Family	Gene	Gene function	Relevant human phenotype association(s)	Gene expression*	Animal model**	Gene constrain metrics (90% CI)***	Patient phenotype
FIN4	*NTRK1*	Ligand for neurotrophins, regulate development of the central and the peripheral nervous systems (Bibel 2000)	Insensitivity to pain, congenital, with anhidrosis (AR) (OMIM #191315)	Expressed in brain (mainly caudate, putamen)	Homozygous lethal (mice; IMPC)	LOF: o/e = 0.43 (0.29—0.66); pLI = 0.88 missense: o/e = 0.87 (0.8—0.94); *Z* = 1.45	Moderate to severe ID, Lennox epilepsy, unclear speech, neuropsychiatric symptoms
FIN21	*SYPL1*	Integral membrane protein present in synaptic vesicles (OMIM #616665)	Paralog *SYPL2* gene has been associated with morbid obesity and depression (Jiao et al. 2015; Shi et al. 2011)	Ubiquitously expressed in neuronal and non-neuronal tissues; high expression in the spinal cord	Behavioral, craniofacial, skeletal, reproductive system abnormalities (mice; IMPC). Mice lacking paralog Sypl2 have been reported to display reduced body weight(Jiao et al. 2015)	LOF: o/e = 0.36 (0.17—0.82); pLI = 0.05 missense: o/e = 0.89 (0.77—1.04); *Z* = 0.45	Metopic suture, delayed neurodevelopment and bone maturation, obesity, panic disorder
FIN23	*ITPR2*	Intracellular calcium release channels (Futatsugi 2005)	AD and AR variants in paralog ITPR1 cause Gillespie syndrome (OMIM #147265)	Ubiquitously expressed in neuronal and non-neuronal tissues	Exocrine dysfunction in ITPR2 or ITPR3 double knock-out mice, difficulties in nutrient digestion, early mortality (Futatsugi 2005)	LOF: o/e = 0.5 (0.42—0.61); pLI = 0 missense: o/e = 0.72 (0.69—0.76); *Z* = 3.71	Neurological and ophthalmological abnormalities, deafness
FIN27	*ZKSCAN1*	Cellular proliferation and differentiation; regulates GABA type-A receptor expression in the brain; GABA is the major inhibitory neurotransmitter in the mammalian brain (Mulligan et al. 2012)	Variants in GABA type-A receptor can cause epileptic encephalopathy and susceptibility to various epilepsy types (Ella et al. 2018)	Ubiquitously expressed in neuronal and non-neuronal tissues	ND	LOF: o/e = 0.12 (0.06—0.32); pLI = 0.97 missense: o/e = 0.67 (0.6—0.75); *Z* = 2.12	Syndromic phenotype with severe ID
FIN32	*ZFR*	Important in axon guidance, RNA transport and localization in neurons (Kjærgaard et al. 2015)	Previously been suggested as a candidate gene in spastic paraplegia (Novarino et al. 2014)	Ubiquitously expressed in neuronal and non-neuronal tissues	ND	LOF: o/e = 0.05 (0.02—0.13); pLI = 1 missense: o/e = 0.52 (0.47—0.57); *Z* = 4.09	Mild ID, slender habitus, neuropsychiatric symptoms
FIN45	*POLR2F*	Encodes a subunit of RNA polymerase II; important in synthesizing messenger RNA in eukaryotes (OMIM # 604414)	Variants in paralog POLR2A cause neurodevelopmental syndromes characterized by profound infantile-onset hypotonia, and developmental delays (OMIM # 180660)	Restricted expression to brain and nerve tissues	Metabolic/immune/hematopoietic system abnormalities (mice; IMPC)	LOF: o/e = 0.42 (0.22—0.88); pLI = 0.01 missense: o/e = 0.73 (0.59—0.9); *Z* = 0.89	Profound ID, infantile spasms, never learned to speak or walk, increased saliva production
FIN47	*DNAH3*	Ciliary and flagellar motility (OMIM #603334)	Suggested candidate gene for ID (Kochinke et al. 2016)	Expressed primarily in long and testis	ND	LOF: o/e = 0.69 (0.6—0.8); pLI = 0 missense: o/e = 1 (0.96—1.03); *Z* = 0.03	Profound ID, congenital hydrocephalus, no speech, nystagmus, dysmorphic ear lobes
FIN-ID3	*ERGIC3*	A component of ERGIC, which mediates the transport from the endoplasmic reticulum to the Golgi (OMIM # 616971)	Suggested candidate gene for ID (Monies et al. 2019)	Ubiquitously expressed in neuronal and non-neuronal tissues	ND	LOF: o/e = 0.46 (0.3—0.75); pLI = 0 missense: o/e = 0.71 (0.62—0.81); *Z* = 1.55	Mild ID, cleft lip
FIN-AIC2	*KIF1B*	transports mitochondria and synaptic vesicle precursors; involved in apoptosis (OMIM # 605995)	AD Charcot-Marie-Tooth disease, type 2A1; susceptibility to tumors (OMIM # 605995)	Preferential expression in brain and skeletal muscle	Homozygous lethal, abnormal embryo size, head shape, limb morphology (mice; IMPC)	LOF: o/e = 0.1 (0.06—0.17); pLI = 1 missense: o/e = 0.68 (0.63—0.72); *Z* = 3.6	Epilepsy, severe ID, coloboma

*Based on the Genotype-tissue expression consortium (GTEx) data (https://www.gtexportal.org/home/), unless referenced otherwise. ** Based on the International Mouse Phenotyping Consortium (IMPC; https://www.mousephenotype.org/), unless referenced otherwise. ***Gene constrain metrics (90% confidence interval), based on the gnomAD database (https://gnomad.broadinstitute.org/): o/e: The ratio of the observed / expected number of loss-of-function or missense variants in that gene. A low o/e value indicates the gene is under stronger selection. pLI: A higher score (maximum 1) indicates more intolerance of protein-truncating variants the transcript appears to be (pLI ≥ 0.9 as an extremely intolerant). Missense Z-score: Higher (more positive) Z scores indicate that the transcript is more intolerant of variation (more constrained)

LOF: Loss-of-function; ND: not determined

### Known variants in known genes

Previously observed pathogenic de novo missense variants were detected in *PPP2R5D* associated with autosomal dominant intellectual disability (MRD35, OMIM #616,355); *ACTB* which causes Baraitser-Winter syndrome (BRWS1, OMIM #243,310); *CYFIP2* which underlies a mild form of early infantile epileptic encephalopathy (EIEE65, OMIM #618,008); and *DYNC1H1* associated with intellectual disability (MRD13, OMIM #614,563) (Figure S1A; Tables 1, 2 & Table S1). Furthermore, two variants were located on the X-chromosome: one in *MED12* underlying FG-syndrome (OMIM #305,450), and a suspected de novo variant in *HUWE1* in a female patient (OMIM #309,590) (Figure S1A; Tables 1 and 2). The phenotypic features are all consistent with earlier publications.

### Novel variants in known genes

We identified 11 novel pathogenic or likely pathogenic variants and four variants of unknown significance (VUS) in known genes in 13 families with neurodevelopmental disorders with ID (Tables 1 and 2).

A young female patient (FIN6-3) was found to have a novel pathogenic frameshift variant [p.(Asp432fs)] in *HNRNPK* which is implicated in AD Au-Kline syndrome (AUKS) (OMIM **#** 616,580). In addition to typical facial features, ID and vesicoureteral reflux, she demonstrated high pain tolerance, and overgrowth on the left scapular region all of which (Figure S1B) are compatible with AUKS.

In family FIN12 a novel and in-frame hemizygous duplication [p.(Pro187dup)] in exon 2 of *ARX* (X-linked) was inherited from the healthy heterozygous mother. The phenotype of the index patient is in agreement with Partington disease with mild ID, dystonic hand movements, and epileptic fits (OMIM **#** 309,510). His brother who has Down syndrome, carries the same *ARX* variant but has no signs resembling Partington syndrome, however, it is unknown whether his trisomy 21 may mask/rescue defects in *ARX.* The variant is rare with no hemizygotes in gnomAD (Table 2; Table S1). Currently, its significance remains unknown.

Study subject FIN14-3 had a novel likely pathogenic de novo non-frameshift deletion in *CTBP1* [p.(Phe53del)]. The subject’s phenotype is characterized by DD/ID, frontal bossing (Figure S1B), hypotonia, difficulties in feeding, psychomotor and growth delay, and ataxic gait. Brain atrophy was found already at one year of age. Interestingly, ophthalmological findings differed from previous cases as FIN14-3 has severe myopia and was operated for cataract at 27 years of age. In the literature, there is only one *CTBP1* variant [p.(Arg331Trp)] which was observed in four unrelated patients who shared features with FIN14-3 (OMIM # 602618). Interestingly, no evidence for tooth enamel defects was detected in FIN-14–3. Both variants are located in the PLDLS-domains of the *CTPB1* and are critically related to transcriptional repression.

A de novo novel pathogenic nonsense variant [p.(Lys620*)] in *CHAMP1* was identified in the DNA sample obtained from FIN20-3 (Figure S1B), which presented with moderate to severe ID, strabismus, constipation, gastroesophageal reflux (GER) and frontal hypoplasia.

For study subject FIN33-3, a de novo likely pathogenic missense variant [p.(Arg1198Ser)] in *RAI1* was identified. This gene underlies Smith-Magenis syndrome (Figure S1B). The phenotypes for FIN20-3 and FIN33-3 were both consistent with previous cases with variants in these genes (OMIM **#** 616579; OMIM # 182290).

FIN36-3 had compound heterozygous variants in *LAMB1* [p.(Glu1668fs); c.2315-28A > G)]. Small cystic lesions in cerebellar hemispheres, white matter abnormalities, and cobblestone cortical malformation of the post-ectopic cortex on MRI (Figure S1B, S2A) resemble previous cases with *LAMB1* variants (OMIM # 615191). Oligohydramnion and enlarged ventricles detected in the fetus during the family’s second pregnancy were likely caused by the same variants. The c.2315-28A > G variant is located in the branch point and may affect splicing.

In family FIN38 a Finnish founder variant [p.(Arg170His)] (Polla et al. 2019) and a novel start loss variant [p.(Met1?)] in *CRADD* were identified. The delayed language development and frontotemporal pachygyria in brain MRI (Figure S2B) were compatible with the earlier findings of *CRADD* (MRT34; OMIM # 614499). The higher minor allele frequency (MAF, 0.0049) of the p.(Arg170His) variant in the Finnish population is consistent with previous reports of this variant as a founder allele in the Finnish population (Polla et al. 2019).

A novel likely pathogenic homozygous missense variant in *P4HTM* [p.(Pro413Leu)] was found in family FIN42 which has two affected sons (Figure S1B; Tables 1 and 2). The variant was not present in the homozygous state in three healthy siblings. Interestingly, *P4HTM* was recently established as a human disease gene that causes HIDEA-syndrome (OMIM **#** 618493). The hallmarks of the HIDEA syndrome are hypotonia, ID, sleeping problems, eye abnormalities, and obesity which were present in both affected siblings.

The elderly male patient FIN49-1 has a unique heterozygous missense variant in *SCN1A* [p.(Met631Val)]*.* Intriguingly, the participant started to move in a crouched gait at 22 years of age, which is a feature recently reported as a characteristic of Dravet syndrome (OMIM # 182389). Given the severe phenotype, he may represent one of the oldest cases of Dravet syndrome found in the literature (74 years).

A likely dual diagnosis was found with a heterozygous missense variant in *TRIO* [p.(Thr1303Ile)] and another in *SON* [p.(Gln2290Pro)] in FIN53-1. Behavioral phenotype (autistic features, hyperactive behavior, obsessions, and aggressions) in the phenotype resemble those brought on by *TRIO* variants (OMIM # 617061) whereas growth retardation and facial features are compatible with the phenotype reported with variants in *SON* (OMIM #617140).

The 15 years old affected male in family FIN-ID4-3 had a hemizygous pathogenic de novo* KIAA2022* nonsense variant [p.(Gln1082*)]. His neurodevelopmental features include hypotonia from early childhood, severe ID, gastroesophageal reflux, and autistic behavior that are compatible with the phenotype underlying pathogenic variants in *KIAA2022* (OMIM # 300912).

The only autosomal dominant variant was found in family FIN-ID8 where a 12 bp deletion variant in *GRIN2A* [p.(Ile151_Ala155delinsThr)] was inherited from the father to his daughter. Both had mild epilepsy during childhood that resolved similar to that previously seen in *GRIN2A* cases (# 245570; FESD). In addition to the *GRIN2A* variant, the daughter has an unknown syndrome (data not shown) that was not solved in this study.

Last, a young female (FIN-ID10-3) presented with apparent multiple disorders. We found a de novo variant in *ANKRD11* [p.(Ala2265fs)] which is in agreement with her phenotype (Figure S1B) and KBG-syndrome (#148050; KBGS). She also has Mayer-Rokitansky syndrome (OMIM #277000; MRKH SYNDROME) for which the cause is still undetermined.

### Phenotypic expansions

We identified variants in five genes, *SAMD9L* (OMIM # 159550; ATXPC), *BCL11A* (OMIM # 617101; DIAS-LOGAN syndrome), *MECP2* (OMIM # 312750; RTT), *MYT1* (OMIM **#** 600379; MYT1) and *ZC3H14* (OMIM # 617125; MRT5) for which we suggest phenotypic expansion (Table 1). The phenotypes were different in severity and/or had partial overlapping features to what previously had been reported (Suppl data; Figure S1C).

The phenotype of the proband with de novo missense variant in p.(Ile908Val) in *SAMD9L* is characterized by moderate ID, clumsiness, and delayed speech development. Variants in *SAMD9L* have been reported to cause ataxia-pancytopenia syndrome (AP) (OMIM # 159550). None of these characteristics were detected in our subject. Due to the extensive phenotypic variability associated with *SAMD9L* variants, more cases need to be identified to properly define the phenotype caused by *SAMD9L* variants.

Variants in *BCL11A* underlie Dias-Logan syndrome, which includes variable dysmorphic features and persistent fetal hemoglobin (fHb) (OMIM # 617101). The phenotype of our proband resembles Dias-Logan syndrome (Figure S1C). However, his fHb is normal (< 1%).

A heterozygous pathogenic frameshift deletion [p.(Val392fs)] in the C-terminal region of *MECP2* was detected in FIN28-3 (Figure S1C). Variants affecting the function of *MECP2* are typically the foundation of classical Rett syndrome. However, the phenotype of the young female here was mild compared to classical Rett or Rett-like syndrome and resembles the phenotype described by Huppke et al. (Huppke et al. 2006). Consequently, we tested X-inactivation and found no evidence of skewed X-inactivation (ratio: 62:38) based on a blood sample. As the variant is in the 3′ region and last exon of *MECP2*, it is predicted to escape nonsense-mediated decay, therefore the milder phenotype might be due to a partially functional protein that is still expressed. The p.(Val392fs) variant was classified as pathogenic (SCV001168944.1) in the ClinVar database, however no phenotypic details were provided.

Last, a de novo missense variant p.(Glu264Gln) in *MYT1* was identified in FIN35-3. Previously, a de novo subtelomeric deletion on chromosome 20 containing *MYT1* and *PCMTD2* have been reported (VCV000058980.1). Both of these genes affect myelination and neural differentiation. Interestingly, these genetic changes share some common phenotypic features including ID, abnormal facial features, lack of speech and communication, and structural abnormalities of the fingers (Figure S1C). Surprisingly, variants in *MYT1* have been identified on the oculo-auriculo-vertebral spectrum (OAVS) in patients who have normal intelligence. FIN35-3 also carried a de novo variant p.(Gly382Val) in *COL9A2* implicated in AR Stickler syndrome and AD epiphyseal dysplasia. It is unclear whether this variant contributes to the skeletal phenotype observed in this patient.

For the female patient FIN-AIC3-3, we identified a homozygous splice region variant c.1177 + 9 T > C in *ZC3H14* (OMIM # 617125; MRT5) of unknown significance. Although this gene has been implicated in non-syndromic AR mental retardation 56, the disorder in our patient is a much more severe multi-system disorder that, based on ophthalmological and brain MRI findings, was resembling Aicardi syndrome. Interestingly, loss of the ortholog of *ZC3H14*, dNab2, in drosophila leads to morphological defects, including those of the eye and displayed severely compromised flight behavior, and poor locomotor activity (Pak et al. 2011). The severe phenotype in the fly more closely resembles the severe phenotype in our patient.

### Alternate inheritance model

Three genes followed a different mode of inheritance than was previously reported for a similar disorder (Table 1). Two of them (*DDX47* and *DHX58)* are members of the DDX/DHX family, which has recently been implicated in neurodevelopmental disorders (Paine et al. 2019). In contrast to previously reported AR variants in *DDX47* and *DHX58* (Figure S1D), both of our cases have de novo variants. Two affected males of families FIN46 and FIN-ID9, both display a severe neurodevelopmental disorder first noted in the newborn period. FIN46-3, with a variant in *DDX47,* has a profound ID, no speech, hypnic jerks, and has been non-ambulatory from seven years of age. FIN-ID9-4, with a *DHX58* variant had severe feeding difficulties, delayed growth, no speech and epilepsy. As a child, he was prone to infections and was extensively studied for metabolic diseases. The third case (FIN7-3) has a mosaic (de novo somatic or gonosomal) variant predicted to impact splicing of *UBA7* (c.1904 + 3A > G). The phenotype was characterized by moderate ID without syndromic features (Suppl information). Previously, a homozygous variant p.(Glu397*) in *UBA7* was reported in a Pakistani family with an AR inheritance pattern (Harripaul et al. 2018). It has been speculated that heterozygosity for this nonsense variant is a risk factor for milder cognitive disability (Harripaul et al. 2018), and it is present in higher frequencies in South Asian populations (gnomAD MAF = 0.0047).

### Candidate variants in potential novel genes

We identified variants of interest in a total of 9 novel candidate ID genes (Figure S1E; Tables 1, 2 and 3; Table S1). Of them, three were autosomal recessive homozygous variants which originated from a sub-isolate of North Eastern Finland where an increased size and frequency in runs of homozygosity were detected (Table 4).

**Table 4:** Runs of homozygosity analysis results

Population	NSEG	Mb	MbA	Fhat1	Fhat2	Fhat3
SAS	44.7 [1.8]	193.5 [11.1]	3.93 [0.10]	0.098 [0.005]	0.106 [0.005]	0.102 [0.005]
EUR	19.5 [1.4]	31.9 [2.7]	1.62 [0.05]	0.037 [0.016]	0.030 [0.010]	0.034 [0.011]
FIN all	24.6 [0.8]	46.8 [2.0]	1.88 [0.04]	0.025 [0.003]	0.035 [0.004]	0.030 [0.002]
FIN Kainuu	26.1 [1.0]	50.9 [2.4]	1.94 [0.04]	0.030 [0.004]	0.033 [0.005]	0.032 [0.002]
FIN Western	24.3 [1.4]	45.1 [4.0]	1.85 [0.11]	0.017 [0.005]	0.049 [0.006]	0.033 [0.005]

NSEG Number of runs of homozygosity (minimum 1 Mb)

Mb Total length of runs (Mb)

MbA Average length of runs (Mb)

Fhat1 inbreeding coefficient 1: Variance-standardized relationship minus 1

Fhat2 inbreeding coefficient 2: Excess homozygosity-based inbreeding estimate

Fhat3 inbreeding coefficient 3: Estimate based on correlation between uniting gametes

brackets indicated standard error of mean

EUR: Non-Finnish European ancestry (outbred); SAS: South Asian ancestry (inbred); FIN: Finnish ancestry

In short, we identified a de novo variant in *NTRK1* also called *TRKA* [mosaic mixture of p.(Tyr757*) and p.(Tyr757 =)] in a child with moderate to severe ID, unclear speech, and Lennox epilepsy (FIN4), a gene important in the development of the central and peripheral nervous system (Bibel 2000) and currently only associated with insensitivity to pain (Indo et al. 1996).

A homozygous missense variant [p.(Cys51Tyr)] in *SYPL1* (OMIM # 616,665), was found in FIN21-3 whose phenotype characterized by metopic ridge and delayed bone maturation (-4.5SD), mild ID, panic disorder, and obesity [body-mass index (BMI = 36)] (Figure S1E). The phenotype strikingly resembles the mouse knockout *Sypl1*^*−/−*^ phenotype characterized by the International Mouse Phenotyping Consortium (IMPC) including abnormal cranium morphology and skeleton, behavioral/neurological features, increased fasting glucose level, and abnormalities in homeostasis/metabolism (Table 3). These data suggest that a metabolic syndrome may belong to the *SYPL1* phenotype. Interestingly, its paralog *SYPL2* has been associated with morbid obesity and depression (Jiao et al. 2015; Shi et al. 2011).

In FIN23-3, a candidate de novo variant [p.(Lys181Glu)] in 1,4,5-triphosphate receptor, type 2 (*ITRP2*) was identified in the sample from the patient, who has a phenotype resembling Gillespie syndrome caused by both AD and AR variants in *ITPR1* (OMIM # 147265). Previously, there was only one variant [p.(Gly2498Ser)] reported in *ITPR2* that causes AR anhidrosis with normal sweat gland in one family (OMIM #106190) (Klar et al. 2014). Thus, the phenotype of FIN23-3 with neurological and ophthalmological abnormalities and deafness differs markedly from the aforementioned phenotype.

FIN27-3, a young male has a severe syndromic ID (Figure S1E). He has a de novo candidate missense variant [p.(Phe434Leu)] in *ZKSCAN1* (OMIM # 601260). This gene has been found to regulate the expression of GABA type-A receptors, the major inhibitory neurotransmitter in the brain (Mulligan et al. 2012).

FIN32-3 is a young female who displays slender habitus, mild ID and neuropsychiatric symptoms has a de novo* ZFR* missense variant [p.(Asp889Glu)] (OMIM # 615635)*. ZFR* has been implicated in axon guidance, neurogenesis, and mRNA transport in neurons (Kjærgaard et al. 2015). It has previously been suggested as a candidate gene for spastic paraplegia (Novarino et al. 2014) and is also a strong candidate gene for ID.

For patient FIN45-3 a previously unknown de novo splice variant (c.294-2A > G) in *POLR2F* was identified (OMIM # 604414). The phenotype was detectable for FIN45-3 as a newborn and manifests as profound ID. Recently, 16 cases of neurodevelopmental syndromes characterized by profound infantile-onset hypotonia, and developmental delays with de novo variants in another RNA polymerase II subunit A gene, *POLR2A* (OMIM # 180660) were described by Haijes et al. (Haijes et al. 2019). In addition, heterozygous mice in the International Mouse Phenotyping Consortium (IMPC) show low circulating albumin levels. Interestingly, a low albumin level was also detected in FIN45-3.

For male patient FIN47-3*,* we identified a heterozygous variant in *DNAH3* [p.(Ile3989Val)], which was absent from the unaffected mother and sibling. Six *DNAH3 *de novo missense variants were found in patients with a neurodevelopmental disorder and ID in the large Deciphering Developmental Disorders (DDD) Study (4293 families) (Deciphering Developmental Disorders Study 2017) and it was suggested as a candidate gene for ID (Kochinke et al. 2016). Unfortunately, the clinical details of these six patients are unavailable. Of notice, several of the novel candidate genes show intolerance towards missense and/or loss-of-function variants (LOF) based on constraint metrics (Table 3), particularly *ZKSCAN1*, *ZFR*, *KIF1B,* and *ZC3H14*.

In family FIN-ID3, we identified a homozygous splice site variant in *ERGIC3* (c.717 + 1G > A) (OMIM # 616,971) in both affected sibs. This gene is important in mediating the transport from the endoplasmic reticulum to the Golgi and has been mentioned as a possible AR ID candidate gene based on a single male patient with growth retardation, microcephaly, learning disability, facial dysmorphism and abnormal pigmentation (Monies et al. 2019).

For the female patient FIN-AIC2-3, compound heterozygous *KIF1B* [p.(Pro689Leu); p.(Pro848Leu)] variants were identified. This gene is highly expressed in neurons, brain, and skeletal tissues and is important in anterograde mitochondrial transport in mammalian neurons (Nangaku et al. 1994; MacAskill and Kittler 2010).

### Structural variants (SVs)

The phenotype of FIN10 is consistent with a pathogenic partial mosaic maternal uniparental disomy (UPD) with a 2.7 Mb deletion on chromosome 21q22.12-q22.2 on paternal chromosome 21, which was identified via microarray analysis. A deletion was found in 22–23% of the cells analyzed from peripheral blood. Although the proportion of the deletion is relatively small, all symptoms—profound ID, absence of speech, epilepsy, microcephaly, growth retardation, and dysmorphic features—are similar to those described in 21q22.12q22.2-deletions which includes *DYRK1A* (Figure S1F). In addition to the *DYRK1A* deletion mosaicism, complex mosaicism of three different homozygosity regions, 21q22.11q22.3 (78%), 21q21.3q22.11 (30%), and 21q21.1q21.3 (15%), was detected resulting from maternal uniparental disomy (UPD) (Figure S4). The region contains also *KCNJ6* underlying Keppen-Lubinsky syndrome (OMIM #614098) characterized by severe ID, seizures and microcephaly. It can have an additional effect on the phenotype. The study has been validated by FISH using a probe close to the DYRK1A region (see KF19-276) (Figure S3A). Parental samples were not available to rule out rare balanced rearrangements involving the 21q22.12q22.2-region using FISH.

Using both microarray analysis and exome sequencing an inherited heterozygous deletion in *NDE1* (16p13.11del) was found in FIN43 (Figure S1F; S2B). The deletion was inherited from the unaffected father. The phenotype (primary and severe microcephaly, partial agenesis of the corpus callosum and simplified gyral pattern) was compatible to what has been previously described (OMIM # 614019). Unfortunately, we were unable to detect the second variant in this gene. The patient of FIN48 has a 106 kb heterozygous deletion at 22q13.33 (Figure S1F; S2C), which was not found in his unaffected mother. The deletion covers *SHANK3* for which 15 C-terminal exons are deleted. The participant’s phenotype is characterized by normal growth, hypotonia, absent speech, severe ID, and autistic features (Figure S1F). He also has high pain tolerance, aggressions, and hand movements resembling choreoathetosis. The phenotype is compatible with the 22q13.3 deletion syndrome (OMIM # 606232; PHMDS).

### Runs of homozygosity analysis

As the Finnish population is an isolated population and only a limited number of homozygous variants were identified, we analyzed the absence of heterozygosity and level of inbreeding in comparison to an inbred and outbred population (Table 4). Overall, the number (NSEG) and size of runs (Mb: Total length of runs in Mb; MbA: Average length of runs in Mb) of homozygosity was higher in the Finnish individuals with ID (FIN) compared to individuals of non-Finnish European (EUR) ancestry (Table 4; p_NSEG_ = 0.003; p_Mb_ = 3.4 × 10^–4^; p_MbA_ = 0.002), which was more pronounced in the Finnish individuals from the North Eastern sub-isolate Kainuu region versus individuals from other regions in Finland (Table 4; p_NSEG_ = 0.014; p_Mb_ = 0.006; p_MbA_ = 0.015). However, the inbreeding coefficients (IBC; Fhat1-3; Table 4), all measures of inbreeding, did not demonstrate excess homozygosity or a higher level of inbreeding in the FIN groups compared to EUR (Table 4; p_Fhat1_ = 0.927; p_Fhat2_ = 0.370; p_Fhat3_ = 0.170). Increased IBCs were only seen in an inbred population from Pakistan compared to both EUR and FIN (Table 4; p_Fhat1-3_ < 1.0 × 10^–15^). Parental consanguinity was only reported in three families, FIN21, FIN-ID3 and FIN-ID9, of them two harbor AR candidate variants. No additional families were found to be consanguineous based on identity-by-descent analysis and KING.

## Discussion

Our study demonstrates that de novo variants are the most common cause of ID in the founder population of Finland. Of the 39 families, known or novel likely pathogenic and pathogenic variants and SVs in previously identified ID-genes were found in 25 families (64%). We suggested a phenotypic extension in five families (13%), an alternate inheritance model in three families (8%), and an abnormal molecular karyotype finding in three families (8%). For a total of 56% of families de novo (or suspected de novo) variants (22/39, including SVs and mosaic variants) were identified. Eighteen (46%) families had de novo variants in known ID genes, which is in line with previously published studies in European populations (Martin et al. 2018). The number of X-linked variants, of them two suspected de novo and three inherited (5/39; 13%) is in agreement with previous studies (de Brouwer et al. 2007). Dual molecular diagnosis was suggested in two families (FIN35, FIN53) (5%) and dual suspected genetic diagnoses in three families (FIN12, FIN-ID8, FIN-ID10) (Table 1). This is consistent with other reports in patients referred to exome sequencing in a clinical setting (Posey et al. 2017). There is evidence that 6% of individuals with autozygosity equivalent to first cousin marriage or greater have a plausibly pathogenic de novo variant in developmental disorders (Deciphering Developmental Disorders Study 2017). This is notable as autosomal recessive variants are known to contribute to ID in populations with high consanguinity (Monies et al. 2017) and isolated populations (Peltonen et al. 1999). Both situations can lead to the absence of heterozygosity (AOH). Although our analysis shows longer stretches of homozygosity exist in the Finnish population compared to mixed populations, there is no excess homozygosity found based on IBC calculations (Table 4). This finding may reflect the distant relationships in the Finnish population traced back to the internal migrations during the sixteenth century (Peltonen et al. 1999; Polla et al. 2019) and that distant relationship reduces the prevalence of ARID. In this study, 5% (2/39) of the families had variants inherited with an AR mode of inheritance which were diagnostic (pathogenic or likely pathogenic), and 15% (6/39) of the families had suspected causal AR variants (VUS) (Tables 1 and 2). This result shows that Finns have a similar or slightly increased contribution of recessive ID-causing variants than mixed European populations (Martin et al. 2018), however, much lower than seen in inbred populations (49%) (Anazi et al. 2017). In fact, previous studies have indicated that recent consanguinity is more important than small population size for detecting a strong effect of AR variants (Mooney et al. 2018), and here, the same trend for ID as for outbred populations is seen, i.e. the majority of variants are de novo.

We identified four homozygous variants which segregated with the disease trait in an AR manner and are either unique to or show enrichment in allele frequency in the Finnish population (Tables 1, 2; Table S1). Three homozygous variants in candidate genes originated from the North Eastern part of Finland where an increased size and frequency in runs of homozygosity were detected (Table 4). First, a homozygous missense variant [p.(Cys51Tyr)] in *SYPL1* (OMIM # 616665), was enriched in the Finnish population in gnomAD. Based on our exome data and additional screening in this sparsely populated region of Finland, we found a carrier frequency of 1:37 suggesting a founder effect for the *SYPL1* variant. No homozygous variants were found in unaffected individuals. In line with several AR disorders that have been discovered in North-Eastern Finland (Peltonen et al. 1999), *SYPL1*, *ERGIC3,* and *ZC3H14* may be novel founder variants in this sparsely populated region of Finland.

The high presence of de novo variants supports the hypothesis of clan genomics, the concept that novel rare variation more significantly contributes to disease in populations, in the development of ID (Lupski et al. 2011). However, in addition to the known Finnish founder variant in *CRADD* [p.(Arg170His)] we identified in family FIN38 (Polla et al. 2019), all the homozygous variants identified were found to be present at a low frequency in gnomAD as well, either exclusive or with a larger frequency in the Finnish population (Table 2). Therefore, some of these might represent older founder alleles enriched due to the unique history of the Finnish population.

We also identified 3 genes which followed a different inheritance model yet still showing a similar disorder (Table 1). *DDX47* and *DHX58* are members of the DDX/DHX family which has recently been implicated in neurodevelopmental disorders (Paine et al. 2019). However, the severe clinical phenotype, present as a newborn, resembles previous cases (Paine et al. 2019). In fact, a majority of the published variants in the DExD/H-box RNA helicase genes have been de novo. There are several examples of both autosomal dominant and recessive inheritance in neurodevelopmental disorders leading to a slight variability in phenotype have been described (Harel et al. 2016). The DDX/DHX family also shows several genes with both mono and biallelic variants suggested to be implicated in neurodevelopmental disease (Paine et al. 2019). The presentation of a phenotype in an AR or AD phenotype is likely related to the impact of the variant on protein function. Due to the LOF tolerance of several of the DDX/DHX genes implicated in neurodevelopment, including *DDX47* and *DHX58,* AD variants are more likely to have a gain-of-function or dominant-negative effect.

The majority of novel candidates also displayed de novo variants. Several of these genes had striking similarities with syndromes associated with a human paralog gene. For example, in FIN23-3, a candidate de novo variant [p.(Lys181Glu)] in 1,4,5-triphosphate receptor, type 2 (*ITRP2)* was identified. Previously, there was only one variant in *ITPR2* reported in OMIM-database [p.(Gly2498Ser)] that causes AR anhidrosis with normal sweat gland in one family (OMIM #106190). A closer look at the phenotype suggests that the clinical features resemble more Gillespie syndrome (Table 1; Suppl information; Figure S1B and S2C) caused by both AD and AR variants in the paralog gene *ITPR1* (OMIM # 147265). The domain structure between *ITPR1* and *ITPR2* seems similar. The p.(Lys181Glu) *ITPR2* variant is located at the N-terminal region (IP3 binding domain), where many of the causative variants are located. The homologous region and phenotype similarities suggests that *ITPR2* may cause the phenotype observed in FIN23-3.

SVs were found in three families (8%). The result is in line with other molecular karyotyping studies in ID (6%-12%) (Moeschler et al. 2014; Cheng et al. 2019). Perhaps the most interesting SV is the complex upd21q22.12-22mat/2.7 Mb del mosaicism that covers *DYRK1A* and *KCNJ6*. The maternal UPD involving the 21q22.11q22.3 region arose mitotically. This is probably a correction mechanism to the deleterious 21q22.12q22.2-deletion originated in the paternal chromosome (Jongmans et al. 2012), and a proportion of the 21q22.12q22.2-deletion and UPD might differ between tissues. *DYRK1A* gene deletion mosaicism has been described earlier at least in five cases (Oegema et al. 2010; Yamamoto et al. 2011), of them, the clinical picture of the patient with the largest deletion (11 Mb) mosaicism was more severe. UPD of chromosome 21 is not known to cause any specific syndrome, but isodisomic regions are risk areas for homozygosity in recessive disease genes. In this patient, the most relevant region is 21q22.11q22.3 presenting 78% homozygosity. Individual cell populations cannot be detected by array analysis, but the homozygous regions are most probably formed independently, presenting UPD of 21q22.11qter (48%), 21q21.3qter (15%), and 21q21.1qter (15%) cell lines. Still, three successive recombination events leading first to UPD (21q22.11q22.3) homozygosity followed by the formation of UPD (21q21.3q22.11) and UPD (21q21.1q21.3) regions cannot be ruled out. The formation of three different homozygosity regions might also cause disruption of the function of the genes located in recombination areas.

In conclusion, our study shows that de novo variants represent the most frequent cause of intellectual disorders in the Finnish founder population. In addition, we expand the phenotypic and genotypic spectrum of several ID genes and present novel candidate genes that could be involved in ID etiology.

## Supplementary Information

Below is the link to the electronic supplementary material.

Supplementary file1 (DOCX 91 KB)

Supplementary file2 (JPG 1363 KB)

Supplementary file3 (JPG 2865 KB)

Supplementary file4 (JPG 2293 KB)

Supplementary file5 (JPG 297 KB)

Supplementary file6 (JPG 2019 KB)

Supplementary file7 (JPG 1244 KB)

Supplementary file8 (PPTX 2534 KB)

Supplementary file9 (JPG 853 KB)

Supplementary file10 (JPG 140 KB)

Supplementary file11 (XLSX 41 KB)

## Data Availability

Variants were deposited into ClinVar under accession numbers: SCV001441481- SCV001441485; SCV001438276.1- SCV001438311.1; SCV001448210.1- SCV001448212.1. The raw whole-exome sequencing data of the affected children and their parents cannot be made publicly available for reasons of individual’s confidentiality. All other in this study-generated data are included in the article (and its supplemental data). Written consent forms of the participating subjects or their legal representatives, alive or deceased, are available upon request. Processed genetic data generated or analyzed within this study are available upon request.
