# Supplementary material for: Exome sequencing reveals predominantly de novo variants in disorders with intellectual disability (ID) in the founder population of Finland
Source: Hum Genet. 2021 Mar 12;140(7):1011–29. doi: 10.1007/s00439-021-02268-1 (PMC8197721; doi:10.1007/s00439-021-02268-1)
Supplement: Supplementary file 1 — Supplementary file1 (DOCX 91 KB) [file 439_2021_2268_MOESM1_ESM.docx]

**SUPPLEMENTAL INFORMATION**

**Exome sequencing reveals predominantly *de novo* variants in disorders with**

**intellectual disability (ID) in the founder Finnish population**

Irma Järvelä^1^, Tuomo Määttä​^2^, Anushree Acharya^3^, Juha Leppälä^4^, Shalini N. Jhangiani ^5^, Maria Arvio^6,7,8,9^, Auli Siren^10^, Minna Kankuri-Tammilehto^9.11^, Hannaleena Kokkonen^12^, Maarit Palomäki^13^, Teppo Varilo^1^, Mary Fang^14,^ Trevor D. Hadley^14^, Angad Jolly^15^, Tarja Linnankivi^16^, Ritva Paetau^16^, Anni Saarela^17^, Reetta Kälviäinen^17^, Jan Olme^18^, Liz M. Nouel-Saied^3^, Diana M Cornejo-Sanchez^3^, Lorida Llaci^19^, James R. Lupski^5,19,20,21^, Jennifer E. Posey^5^, Suzanne M. Leal^3^, Isabelle Schrauwen^3^

**Supplemental methods**

*Exome sequencing and bioinformatic analyses*

The sequencing depth of on target regions was >50x for each sample. Filtered high-quality reads were aligned to the human reference genome (GRCh37/Hg19) using Burrows-Wheeler Aligner-MEM (BWAv0.7.15) (Li and Durbin 2009). Duplicate reads were marked using Picard-tools (v2.5.0). Insertion/deletion (InDel)-realignment and base quality score recalibration was performed with the Genome Analysis Toolkit (GATK) (v3.7). Single nucleotide variants (SNVs) and InDels were called using GATK HaplotypeCaller (https://gatk.broadinstitute.org). As a quality control, heterozygosity level, gender, maternity, and paternity were confirmed for each pedigree member using the relatedness functions in VCFtools (v0.1.13), which includes Kinship-based Inference (KING), and identity-by-descent IBD calculations in plink(v1.90) (Chang et al. 2015). Variant annotation and filtering were performed using ANNOVAR (Yang and Wang 2015) and custom scripts. Criteria for filtering included the following: exonic and splice region from intron-exon boundary were retained; several inheritance models were considered depending on the pedigree, that included but not limited to *de novo*, AR, and X-linked. Variants with a predicted effect on protein function or pre-mRNA splicing (nonsense, missense, frameshift, splice region, etc.) with a population specific minor allele frequency (MAF) of <0.01 (for AR) and <0.0005 [for autosomal dominant (AD) and X-linked] in all populations of the Genome Aggregation Database (gnomAD; https://gnomad.broadinstitute.org/) were retained. Bioinformatic prediction scores were annotated from dbnsfp35a and dbscSNV1.1 to evaluate missense and splice site variants respectively (Challis et al. 2012). For families FIN-ID3, 4, 8, 9 and 10, SNV/Indel variants were called using Atlas2 (Bainbridge et al. 2011), and variant annotation was performed using the in-house developed Cassandra pipeline (Krumm et al. 2012).

Copy number variants (CNV) were assessed using Copy Number Inference from Exome Reads (CoNIFERv.0.2.2) (Gambin et al. 2017). The database of genomic variants (Dgv) and gnomAD were used to identify rare CNV variants (MAF) of <0.01 (for AR) and <0.0005 (for AD and X-linked). For families FIN-ID3, 4, 8, 9 and 10, CNVs were assessed from exome variant data using HMZDelFinder to detect intragenic homozygous CNVs and XHMM (Fromer and Purcell 2014).

*X-inactivation analysis*

X-inactivation analysis was performed using genomic DNA obtained from peripheral blood using the human Androgen Receptor (HUMARA) gene assay. In short, PCR amplification of a short tandem repeat within the first exon of the HUMARA on the X-chromosome was done with and without methylation-sensitive HpaII restriction enzyme treatment to determine the methylation status of parental alleles.

*Molecular karyotyping*

A chromosomal microarray was performed from DNA extracted from a lymphoblastoid cell line using 50mer oligochip (HumanCytoSNP-12 (v2.1) Illumina), which contains ~ 300 000 (300K) oligonucleotide probes spaced 10 kb apart genome-wide and at 6 kb in over 250 clinically significant regions. The analysis was done using GenomeStudio -, KaryoStudio- (Illumina). and BlueFuseMulti (BlueGnome) programs. The HumanCytoSNP-12chip can detect copy number changes >30 kb in cytogenetically relevant regions and >200kb in other areas of the genome. Abnormal results are reported according to ISCN 2016 using annotation GRCh37/hg19; copy number changes considered as normal variation are not reported. In addition to copy number changes regions of SNP homozygosity can also be detected (< 10Mb regions are not reported). Limitations: analysis cannot detect balanced rearrangements (e.g. translocations, inversions), gene variants and low-level mosaicism.

FISH-analysis was done both from uncultured (n= 200 interphases) and cultured (n=300 interphases, 25 metaphases) peripheral blood lymphocytes using probe mix detecting DNA-sequences from *DYRK1A* gene region (RP11-777J19, chr21:38719388-38887895, hg19, Orange 5-TAMRA dUTP, Empire Genetics) and from 21q21.1 control region (RP11-687D14, chr21:16993294-17155825, hg19, Green 5-fluorescein, Empire Genetics). The deletion mosaicism was confirmed by fluorescent in situ hybridization (FISH) in which the deletion cell line was detected in 23.5% of uncultured and 23 % of cultured interphases and 24 % of metaphases.

**Clinical description of patients**

*Known gene – known variant*

**FIN3-3** is a 14-year-old male, and the II/4 child of the family. He was born at 41 +2 weeks of pregnancy and was delivered normally. At birth his weight was 3155 grams (g), height 49 centimeters (cm) and head circumference 36.5 cm. He had neonatal hypoglycemia, foramen ovale, patent ductus arteriosus (PDA), and a small ventricular septal defect (VSD). A developmental delay was noticed soon after birth along with muscle hypotonia and nystagmus. A partial, isolated growth hormone deficiency was diagnosed at 2 years of age. He learned to walk at 13 years of age. His only word is mother. At the age of 13 years his height was -3 standard deviations (SD), weight -15%, and head circumference 61 cm (+5.87 SD). His phenotype is characterized by severe ID which is progressive in nature. He has a prominent forehead, narrow parietal regions, an open mouth, a high palate, small teeth, thick lips, a sunset gaze, nystagmus, strabismus, anisometropia, and -8,5 dioptres l.dx (Fig.1A). Both eyes have been operated due to cataracts. Hitting his eyes has caused bleeding in the corpus vitreum that led to blindness. Due to his restless behavior and aggressive bursts, he needs continuous care. His brain MRI, molecular karyotype, and muscle biopsy are normal with the exception of macrocephaly. A paternal sample was unavailable to confirm that the variant is *de novo*, however, the same variant has been previously reported as a *de novo* known pathogenic missense variant p.(E198K) in *PPP2R5D* (OMIM **#** 616355). His phenotype is consistent with *PPP2R5D*-related disease.

**FIN11-3** is a 24-year-old female who is the V/7 child of the family. At birth her weight was 3345g, height 38 cm, head circumference 33 cm, and Apgar score of 9/10/10. At 8 months of age she was evaluated due to delayed psychomotor development, problems in feeding, and obstipation. She learned to walk at 23 months of age. In EEG, slow-wave irritation was focused on the right frontal region but no epileptic fits were observed. She is sensitive to sensory stimuli, smells, and tastes. At 5 years of age she started to have migraine attacks. Her behavior was characterized by anxiety and repetition. She started to get panic attacks at 9.5 years of age. She has obtained special education. Scoliosis was diagnosed at 10 years of age. Due to unclear speech, she has obtained speech therapy. She learned to read at 13 years of age. Her adult height is 150 cm (mother 150 cm and father 161 cm). She has mild ID. Her brain MRI, karyotype, and urine metabolic screen are normal. Her facial features include a tented upper lip, and overall thick lips (Figure 1a). She has truncal hypotonia. She needs help in everyday tasks. A *de novo* missense variant p.(E665K) in *CYFIP2* (cytoplasmic FMRP-interacting protein 2 gene) was identified*.* The parents do not carry the variant. *CYFIP2* variants usually cause AD early infantile epileptic encephalopathy, ID, and muscular hypotonia. One case with the same variant, p.(E665K), has been previously reported have a milder phenotype with no epilepsy (OMIM **#** 618008). The phenotype of patient FIN11-3 is consistent with this finding.

**FIN17-3** is a 21-year-old male, and the II/3 child of the family. His fetal extrasystoles were controlled after 34 gestational weeks. He was born at 41 weeks of pregnancy with a weight of 4030g, length of 53cm, and head circumference of 38 cm. After the birth he breathed with difficulty, was hypotonic, and had an Apgar score of 2/7/8. His crying was weak and high-pitched. As a newborn, a prominent forehead, small and low-set ears, and 5 fingers overlapping 4 fingers were detected. He has been studied due to delayed psychomotor development. He learned to walk at 22 months. He had several febrile seizures that started at 2 years and 7 months of age. In his EEG, focal irritation was present. Additional features include pes planovalgus l.a., umbilical hernia, and constipation. After 4 years of age, he was diagnosed with delayed speech development and dyspraxia. He has obtained physical and speech therapy. His ID is mild. His facial features are characterized by square-shape, open mouth, small ears, and he has wide and flat thumbs and halluces. His karyotype and urine metabolic screen were normal. In the brain MRI, enlarged cisterna magna were present. As an adult he has unclear speech, obsessions, and a panic disorder. A known pathogenic hemizygous variant p.(R961W) in *MED12* underlying Opitz-Kaveggia syndrome (OMIM **#** 305450**)** was found. The mother is a healthy carrier of the variant. The father does not have the variant. The patient’s clinical findings are compatible with Opiz-Kaveggia-syndrome.

**FIN24-3** is 14-year-old female, the II/2 child of the parents and the fourth child of the mother. During the last trimester of pregnancy, an enlarged intestine and hydramnion were detected. She was born during the 36^th^ week of pregnancy, weighing 2225 g, and measuring 43 cm tall. She underwent surgical correction for congenital duodenal atresia after birth. An apple peel deformation was found in the intestine during operation. Severe congenital sensorineural deafness was diagnosed at one year of age. She learned to walk at 17 months of age. Her height has developed at -3 SD curve, and her head circumference at -1 SD. At 2 years of age she developed absence seizures. Her EEG and karyotype are normal. Her facial features are characterized by hypertelorism, epicanthus, large mouth, broad nasal bridge, high and narrow palate, low-set ears and short, and webbed neck (Fig.1a). In an ophthalmological examination, chorioretinal coloboma was found. She has moderate ID. In the brain MRI, pachygyria was detected. At the age of 14, a bicornuate uterus was found at the gynecological examination. She goes to a school for hearing-impaired children. She cannot read and has motor clumsiness. The patient has a known pathogenic missense variant p.(S348L) in *ACTB* (OMIM # 243310**)** known to underlie Baraitser-Winter syndrome. The patient’s phenotype is consistent with this syndrome.

**FIN37-3** is a 14-year-old female patient, the only child of the family. She was born by cesarean section at 39 weeks of pregnancy. At birth her weight was 2550 g, height 43 cm, head circumference 33 cm, and an Apgar score of 9/9. She was hypotonic and had hip luxation as a newborn. Infantile spasms and hypsarrythmia started at 7 months of age. In the brain MRI, frontotemporal pachygyria and lissencephaly were detected. She has severe ID, hypotonia and scoliosis. She communicates with gestures and vocalizations and cannot walk. A previously reported *de novo* variant p.(R1567Q) in *DYNC1H1* was identified that is compatible with her phenotype (OMIM **#** 614563).

**FIN52-2** is a 53-year-old female who presented with hypsarrythmia at 7 months of age. She learned to walk at 3 years of age and learned to speak her first words at 5 years of age. She has severe ID. A heterozygous, known pathogenic variant p.(R3070C) in *HUWE1* was identified. The variant was not present in the healthy mother. Paternal sample was unavailable. Thus, the variant is likely *de novo*. Her facial features are characteristic with *HUWE1* syndrome (Fig. 1a) (OMIM # 309590).

*Known gene – novel variant*

**FIN6-3** is a 10-year-old female, and the III/5 child of the family. The first sign of her disease was poor control of her head which was noticed at 3 months of age and concluded as congenital muscle hypotonia. Her muscle and skin biopsies were normal. Her growth is somewhat delayed (height -2 SD). She learned to walk at 4.5 years of age. She can speak some words and has learned to use signs and pictures to express herself. Her skin is thickened in the region of the left scapula (Fig. 1a). Of notice, her pain tolerance is high, which may cause dangerous situations. The brain MRI displayed cortical dysplasia, a 3 cm region of heterotopia in the white matter, and a neuronal migration defect. She has vesicoureteral reflux grade III. She has had frequent respiratory infections. Her facial features are characterized by a prominent nasal ridge, hypoplastic alae nasi, open, downturned mouth and sparse lateral eyebrows (Fig.1b). Her muscles are weak in her lower extremities, and she uses splints to support independent walking. A novel frameshift variant p.(D432fs) in *HNRNPK* underlying Au-Kline syndrome was identified. Her phenotype is in agreement with Au-Kline syndrome (OMIM **#** 616580). 21-hydroxylase deficiency and long QT have been found in the maternal side of the family. These diseases have been excluded in the patient.

**FIN12-2**is a 43-year-old male, and the youngest of the three sons in the family. He was born at 42 weeks of pregnancy, weighing 4010g, 50 cm tall, and with a head circumference of 37 cm. He had bilateral clubfoot at birth. He learned to walk at 19 months of age and spoke first words at 24 months of age. He had an operation for retention testis at 2 years of age.  Features of mild ID, dysarthria and motor clumsiness were notable during childhood. Between the ages of 14 to 21, he had rare epileptic seizures. In EEG, generalized slowing and in EMNG diffuse polyneuropathy were detected. In the brain MRI, unspecific white matter changes in near lateral ventricle on the left side were found. AED medication was tapered at the age of 21. After the age of 25, the condition started to progress. At the age of 43, he has severe dysarthria, dystonic hand movements-mainly on left side in arms and hands in action and in rest, head nodding, sensations in the throat and skull, and almost constant tremor, and his facial expressions are scarce. His gait is slow and clumsy. He has slight spasticity in his joints, especially in his ankles. Tonic pupillae, nystagmus, strabismus and chalazion were detected during his ophthalmological examination. His hearing is normal. He has panic attacks and insomnia. His clinical features are compatible with Partington disease (OMIM **#** 309510).  A novel hemizygous variant of unknown significance p.(P187dup) in *ARX* was found in the patient and his brother (affected by Down syndrome). His mother was a healthy carrier of the variant. The oldest brother, who is healthy, did not carry the variant.  Of note, the father had an unknown progressive neurological disease, however this study was unable to assess his DNA.

**FIN14-3** is a 35-year-old male, and the III/3 child of the family. He was born at 39+2 weeks of pregnancy at 2880g. Muscle hypotonia and bradycardia (60-80/min) were detected at birth. His muscle biopsy was normal. He has severe psychomotor and growth delay, frontal bossing (Suppl Fig.1b), ureter reflux grade IV, and small testes. Cortical atrophy was detected on brain CT at one year of age. The patient learned to walk at 5 years of age and can speak some words. As a toddler he moved by rolling himself forward. His gait has been ataxic and he needs a wheel chair. He learned to eat by himself at 6 years of age. He needs a lot of help in everyday tasks. His EEG is abnormal. He has strabismus and myopia -8.5/-5 diopters. His cataract was operated on at 27 years of age. His adult height is 149 cm and head circumference 58 cm. His behavior contains rituals. A *de novo* non-frameshift indel p.(F53del) in *CTBP1* was found (OMIM # 602618).

**FIN20-3** is 19-year-old male, and the I/2 child of the family. He was born at 38 weeks of gestation after a normal delivery. Birth weight was 3320g, height 50 cm, and head circumference 33 cm. He was hypotonic at birth. His psychomotor development has been delayed. He started to walk at 2 years of age and can speak some words. He has moderate to severe ID, strabismus, constipation, and gastroesophageal reflux (GER). Frontal hypoplasia was detected in his brain MRI. His facial features include a long face, short philtrum, orofacial hypotonia, thin and tented upper lip, everted lower lip, pointed chin, and deep-set ears (Fig.1b). A *de novo* nonsense variant p.(K620*) in the *CHAMP1* gene was identified which is pathogenic and explains his phenotype (OMIM **#** 616579). The variant is absent from gnomAD.

**FIN33-3** is 12-year-old male, and the only child of his parents. He spoke his first words at one year of age and learned to walk at 1.5 years of age. He was very restless between 1-6 years of age. He has mild ID, delayed speech development, and clumsiness. He has a broad, square face (Fig.1b). His brain MRI and EEG are normal. He has repetitive behavior and a loud, coarse voice. His behavior is challenging as he has a short attention span, hyperactivity, jamming and self-destructive behaviors including tearing his nails until bloody, and trichotillomania. His emotions are rapidly changing from shyness to aggression. A novel *de novo* missense variant was identified in *RAI1*, p.(R1198S). The parents do not have the variant. *RAI1* is known to cause Smith-Magenis-syndrome. The facial and behavioral features are compatible with Smith-Magenis syndrome (OMIM # 182290).

**FIN36-3** is a 30-year-old female who was born after an uneventful third pregnancy. Her birth weight was 2910g, and height was 47.5 cm. She has one healthy brother. The second pregnancy was terminated at 21 weeks due bleeding and oligohydramnion. At autopsy the fetus was found to have enlarged ventricles in the brain. The index patient presented with muscle hypotonia and strabismus at 3 months of age. Non-specific myopathic changes were detected in the muscle biopsy. During the ophthalmological examination, small opacities were detected in both lenses. She learned to walk at 1 year and 10 months of age and to also speak short sentences. At 1.5 years of age, growth retardation was detected, and epileptic fits appeared (Suppl Fig.1b). In the EEG, generalized beta/wave activity was demonstrated. At 2 years and 4 months of age, the brain MRI showed hypoplastic corpus callosum, brain stem and vermis. In addition, mild hypoplasia and small cystic lesions in cerebellar hemispheres, dilated lateral ventricles, and symmetric white matter abnormality were found. Cobblestone cortical malformation with postectopic cortex lining the lateral ventricles is observed (Suppl Fig. 3A). Dentate nuclei were hyperintense and optic nerves atrophic (not shown). Based on her tripping and stumbling she was suspected to have a transient visual field defect. Her psychomotor development was delayed, and she went to a special school. She has moderate ID. Her adult height is 154 cm (-2 SD). She was diagnosed with scoliosis at 11 years of age. Previously, sequencing of exons from 2 to 11 in *LIS1*, the coding regions of *FKRP* (OMIM # 607155), *MDC1C* (OMIM # 606612), the Finnish founder variants underlying muscle/eye/brain disease *MEB* (OMIM # 253280) and *CRADD* (OMIM # 614499) showed negative results. Via ES we found compound heterozygous variants in *LAMB1* p.(E1668fs) + c.2315-28A>G (splicing branch point). Her phenotype resembles the phenotype underlying *LAMB1* variants (OMIM # 615191). The brother did not have any of the variants.

**FIN38-3.** She is an 8 year-old female patient I/2 child of the family. The younger sister is healthy. Her ancestors originate from Swedish-speaking region in Western Finland. Pregnancy and delivery were normal, birth weight 4290g, height 54 cm, head circumference 36,5 cm. Mother’s brother and father had dyslexia. She learned to walk at 20 mo of age. At 3 years of age delayed speech development was noticed and speech therapy started. In the follow-up, delay in psychomotor development was detected. ECG is normal. She has no epilepsy. At 5 years of age MRI showed thick cortex with broad gyri compatible with pachygyria and unspecific punctate white matter lesions (Suppl Fig. 3B). In the right cerebral region, a small convoluted hemorrhage was found. She had normal molecular karyotype. At 8 years of age, attention deficit disorder was detected and methylphenidate started. Mild ID is not excluded. Due to learning difficulties at school she needs strong support. She was found to be compound heterozygous for the variants in CRADD; in one allele a novel variant c.2T>C:p.(M1?) and on the other allele the Finnish founder variant c.509G>A:p.(R170H) were identified.

**FIN42:** There are two affected males in the family. **FIN42-3** is 20-year-old male, and the I/5 son of the family. He was born at 41+3 weeks of pregnancy and had an Apgar score of 8. His birth weight was 3600g, height 49 cm, and head circumference 37 cm. As a newborn, marked generalized muscle hypotonia, strabismus and exophoria were detected. Focal seizures started at 5 mo age, mostly at night. Brain MRI was normal. He was seizure-free from the age of 5 years and his antiepileptic medication was discontinued at the age of 14 y. He learned to walk at the age of 3.5 y and started to speak some words after 4 y of age. He now speaks simple sentences but the speech is unclear. Due to severe ID he has obtained special education. He has always had poor sleep. His gait is unsteady. As an adult due to an uncontrolled appetite his weight increased (height 175 cm, weight 112 kg, BMI 36). He used to bite his fingernails. He works in a shelter for persons with intellectual disabilities.

**FIN42-7** is a 12-year-old male, the youngest of the five brothers in FIN42 family. He was also evaluated at 4 mo of age by pediatric neurologist due hypotonia and strabismus (exophoria). His epileptic seizures started at the age of 5 mo. He was treated with the antiepileptics valproate and topiramate. He learnt to walk at 3.5 years of age. His phenotype is more severe concerning language development as he does not speak. He communicates mostly by gestures. Due to profound ID he has had special education in school. He had 90 ° kyphosis in his neck and thoracic scoliosis. Due to uncontrolled appetite he has also been obese since childhood. At the age of 12 years his height is 167 cm, weight 73 kg and BMI 26. He needs help in everyday tasks, except eating. He likes music and as an autistic feature he likes to watch spinning items. His mood changes easily from happiness to sadness. A novel homozygous missense variant chr3: 49043570 C>T; p.(P413L) in exon 8 of *P4HTM* (OMIM # 618493) was found in both affected sons (Suppl Fig. 1A; Table 1 & 2). The parents and two brothers (FIN42-4, FIN42-5) were healthy carriers (C/T) and one brother (FIN42-6) was homozygous (C/C) for the variant.

**FIN49-1** is 74-year-old male with profound ID. His epilepsy started when he was 6 months old. He learned to walk at 3.5 years of age. He has no speech. His facial features are characterized by a broad nose, thick hair and eyebrows, and a long philtrum (Suppl Fig. 1b). He has had absence seizures starting at 5 years of age. As an adult, generalized tonic-clonic seizures has been prevalently medicated with hydantoin and lamotrigine. He started to move in a crunching position at 22 years of age. He is restless and has aggressive bursts. A rare heterozygous variant p.(M631V) in *SCN1A* was detected. His phenotype is compatible with classical Dravet syndrome (OMIM # 182389). His sister does not have the variant, and samples from the parents were unavailable. There is one individual heterozygous for the same variant in gnomAD. This could be explained by high variability of the clinical phenotype of Dravet syndrome.

**FIN53-1** is a 72-year-old female, and the I/4 child of the family. Data about her childhood is scarce. Her adult height is 144 cm (-4 SD), and her head circumference is 52.5 cm. Her motor and linguistic skills are within normal range. She has moderate ID. Her behavior is characterized by autistic features, obsessions, hyperactivity, and aggressive bursts. She does not have epilepsy, and her vision is normal (Suppl Fig. 1b). Two unique heterozygous missense variants were identified. A missense variant p.(T1303I) in *TRIO*, and p.(Q318P) in *SON* (Table 1 and 2), both absent from gnomAD and the unaffected brother. Her behavioral manifestations resemble the phenotype reported in MRD44 (OMIM # 617061) suggesting a variant in *TRIO.* Her short stature and facial features are compatible with that reported for individuals with variants in *SON* (OMIM #617140).

**FIN-ID4-3** is a 15-year-old male, and the I/2 child of the family (Suppl Fig.1b). He has severe ID. His birth weight was 3740g, height 56cm, head circumference 36cm, and Apgar scores of 7/9/10. He was diagnosed with hypotonia at 4 months of age by a child neurologist. His MRI is normal. He learned to walk at 21 months of age, and he speaks in short sentences. He has been diagnosed with gastroesophageal reflux. He is restless and has aggressive bursts. His absence seizures started at 11 years of age, but his EEG is normal. Until now he has had enuresis nocturna. His adult height is 172 cm, head circumference 56 cm (+ 1SD) (at 11 years of age). *FMR1* repeat expansion testing is negative, karyotype is normal. A novel, *de novo* hemizygous variant p.(Q1082*) in *KIAA2022* was identified in the child. A paternal sample was not available for the study, and the variant was not detected in DNA extracted from a peripheral blood sample from the mother, although we cannot rule out the possibility of gonadal mosaicism. The phenotype of the patient is fitting with the previous findings underlying *KIAA2022* variants (OMIM # 300912).

**FIN-ID8-3** is a 21-year-old female, and the I/2 child of the family. Her birth weight was 2995g, height 48cm, and head circumference 33 cm. Her adult height is 174 cm (+1 SD) (father 187.5 cm and mother 165.5 cm). Her head circumference is 52 cm. She has moderate ID, short philtrum, thin and dysmorphic fingers with a brachydactylic 4th finger and arachnodactylic 5th finger on the left, and slender habitus (Suppl Fig. 1d). Her brain MRI is normal. She can read and write satisfactorily, and her speech and walking are normal. She can use a computer and a mobile phone but needs help in understanding the value of money. She needs support in everyday tasks. She had benign partial epilepsy between 4 -17 years of age. Her father also had epilepsy between 7-15 years of age. He is healthy. The epilepsy phenotype is consistent with having a paternally inherited variant in *GRIN2A* (OMIM # 245570). The cause of the unknown syndrome of FIN-ID8-3 was not identified in this study.

**FIN-ID10-3** is a 23-year-old female, and the II/2 child of the family. She was born by caesarean section. Her birth weight was 3070g and height 47 cm. He adult height is 147 cm (-3.5 SD). She learned to walk at 16 months of age. At 2 years of age, she was diagnosed with delayed speech development, restlessness, hyperkinesia, and difficulties to concentrate. She was additionally diagnosed with dysphasia. At 2.5 years of age, her brain MRI revealed enlarged cisterna magna, and a mild Dandy-Walker variant. At 3 years of age she was diagnosed with a urinary infection, and grade III ureter reflux and agenesis of the vagina and uterus were found. Her kidney ultrasound was normal. Her karyotype was normal, 46, XX. The agenesis of the vagina and uterus as well as the mild Dandy-Walker variant are compatible with the Mayer-Rokitansky syndrome.   In addition, at 4 years of age she was diagnosed with coeliac disease. She learned to speak at 5 years of age. She has moderate ID. She underwent surgical correction of strabismus at 8 years of age. She had normal puberty, severe acne, and normal testosterone levels. She has macrodontia and a low hairline (Suppl Fig. 1b). Her personality is shy, and she has rapid speech. She is working part-time in a kitchen. A *de novo* variant p.(A2265fs) in *ANKRD11* was identified. Moderate ID, short stature, macrodontia, strabismus, low anterior hair line, prominent ears, poor concentration, hyperactivity, and anxiety confirm KBG syndrome as the second rare disease in the patient (OMIM # 148050). KBG-syndrome has rarely been reported in females.

*Known gene – phenotypic expansion*

**FIN5-3** is 39-year-old male, and the II/2 child of the family. He has moderate ID, clumsiness and delayed speech development. His head circumference is 62 cm and height 182.5 cm. His gait is normal, and he does not have ataxia. His IQ has been studied and shows WISC 41 and Merrill-Palmer 68. His brain CT and MRI are normal. There is some irritative activity in EEG, but no epilepsy, and his karyotype and metabolic screen are normal. His hernia inguinalis has been surgically corrected. He walks long distances. He has good humor, is interested in other people, and likes music. No hematological findings including pancytopenia have been detected. A *de novo* missense variant p.(I908V) in *SAMD9L* was classified as likely pathogenic (OMIM # 611170). Previously, autosomal dominantly inherited and *de novo* missense variants have been reported in *SAMD9L* which cause ataxia-pancytopenia (AP), characterized by ataxia, microcephaly, and pancytopenia, with variable age of onset and expressivity of features. None of these characteristics were detected in our subject. However, neurological abnormalities are variable, slowly progressive and can include speech abnormalities and start with a clumsy gait and coordination. For some cases with *SAMD9L* variants, the onset of symptoms starts at an older age. As the phenotypic variability associated with *SAMD9L* variants is great, more cases need to be identified in order to properly define the phenotype caused by *SAMD9L* variants.

**FIN8-3** is 24 a year-old male who is the III/3 child of the family. He was born after a normal delivery at 41+6 weeks of pregnancy with a birth weight 4070g, a length of 55 cm, head circumference of 37 cm, and Apgar scores of 9/9/10. He learned to walk at 14 months of age. He was diagnosed with language delay at 3 years of age and has obtained special education. His adult height is 191cm, weight 118 kg and head circumference 60 cm. As an adult he speaks some words only with his mother. He has moderate ID. His facial features include down slanting palpebral fissures, strabismus, deep set ears, a thin upper lip, and an everted lower lip. His fine motor development is delayed. He has joint laxity. His brain MRI, EEG, and urine metabolic screen have been normal. His behavior is characterized by autistic features, a short attention span, and aggressive bursts. A likely pathogenic *de novo* variant [p.(T326K)] in *BCL11A* (OMIM # 606557) was detected. Variants in *BCL11A* underlie Dias-Logan syndrome, and includes variable dysmorphic features and persistent fetal hemoglobin (fHb). Our participant has facial features, moderate ID and severe language delay which resemble Dias-Logan syndrome (Figure S1C). He has also thin long fingers (Figure S1C). However, his fHb is normal (<1%).

**FIN28-3** is 18-year-old female, and the only child of the family. She learned to walk at one year of age. Her speech delay was noticed at 3 years. She went to a special school. Her adult height is 160 cm and weight 80 kg. Her ID is moderate. She can understand simple text and write short sentences. She is social and likes to do handicrafts. Changing to a new school caused a strong stress reaction characterized by excessive sweating and anxiety that lasted for several months. Later, her health has improved and remained good. She has not had epilepsy nor scoliosis. A *de novo* heterozygous pathogenic frameshift deletion [p.(V392fs)] in the C-terminal part of *MECP2* was found. Variants affecting the function of *MECP2* are typically the foundation of classical Rett syndrome. However, her phenotype is mild and she has no hand stereotypies characteristic to the classical Rett syndrome (OMIM # 300005). Due to her mild phenotype, we tested X-inactivation and found no evidence of skewed X-inactivation (ratio: 62:38) based on a blood sample. As the variant is in the 3’ region and last exon of *MECP2*, it is predicted to escape nonsense mediated decay, therefore the milder phenotype might be due to a partially functional protein that is still expressed. To date, several cases representing mild Rett syndrome have been described with the variant located in the last exon of *MECP2* (Bebbington et al. 2010). The variant has previously been deposited in the ClinVar database and classified as pathogenic (SCV001168944.1), however no phenotypic details were provided.

**FIN35-3** is a 17-year-old female, and the III/3 child of the family. She was born at 39+2 weeks of gestation. Her birth weight was 4865g, height 53 cm and head circumference 37.5 cm. She learned to walk at one year of age. At 2 years of age she was evaluated because of her increased head circumference (+2 SD), delayed speech development, clumsiness and restless behavior. Her brain MRI, EEG, and karyotype were normal. Her adult height is 160cm (mother 157 cm and father 174 cm), she has moderate ID, delayed speech development, short fingers and enuresis nocturna. Her urological examinations were normal. Her personality is shy, and she has been very dependent on her mother. Changes in everyday life are difficult for her. A *de novo* missense variant p.(E264Q) in *MYT1* gene (OMIM # 600379) was identified in WES. Another likely pathogenic *de novo* missense variant p.(G382V) in *COL9A2* (OMIM # 120260) was also found. Previously, several individuals with intellectual disability and a *de novo* subtelomeric deletion on chromosome 20 that contain *MYT1* and *PCMTD2* (Kroepfl et al. 2008) have been identified. Both of these genes affect myelination and neural differentiation. Interestingly, the phenotype includes mild to severe ID, abnormal facial features, lack of speech and communication, and structural abnormalities of the fingers (short metacarpals, aplasia of middle phalanx, and syndactyly) resembling the phenotype of FIN35 (Figure S1C). In addition, variants in *MYT1* have been reported in the oculo-auriculo-vertebral spectrum (OAVS) in patients who have normal intelligence. In addition to the variant in *MYT1*, we also identified a *de novo* p.(G382V) in *COL9A2* classified as VUS. *COL9A2* is implicated in AR Stickler syndrome and AD epiphyseal dysplasia.

**FIN-AIC3-3** is a 16-year-old female, and the only child of the family. She was born to a 28-year-old mother. The mother had one spontaneous abortion before this pregnancy. In an ultrasound at the 35 weeks, hydrocephalus, and a cyst in corpus callosum were detected. The individual was born at week 40+5 by cesarean section due to a delayed delivery. She had an Apgar scores of 9/9, a birth weight of 3900 g, height of 52 cm (+0.9 SD), and a head circumference of 37.7cm (+2 SD). In the brain ultrasound performed when she was a newborn, agenesis of the corpus callosum was found. The brain MRI revealed multiple malformations including deformed ventricles, with heterotopia at left ventricle, wide occipital horns, cysts in the midline and hindbrain, plexus papillomas, and coloboma in the right orbit suggesting Aicardi syndrome. On ophthalmological examination, strabismus on the right, nystagmus, oedema and round white spots in retina were found. Infantile spasms appeared at 4 months of age that changed to Lennox Gastaut -type epilepsy at 6 years of age. Due to increased ventricle size ventriculoperitoneal shunt was set. Recurrent infections starting at 5 years of age. She has personal facial features, profound ID, no speech, spasticity, severe scoliosis, and aseptic necrosis in the right hip. Her karyotype is a normal 46, XX. The molecular karyotype revealed at 14q21.1q21.3 a ~12.2 Mb homozygous region suggesting UPD. Using two markers biparental inheritance was confirmed. A homozygous splice region variant (c.1177+9T>C) in *ZC3H14* (OMIM # 613279) was identified*.*

*Alternate inheritance model*

**FIN7-3** is a 41-year-old male, and the III/3 child of the family. The pregnancy and delivery were normal. His birth weight was 3700g, height 51 cm, and Apgar scores 10/10. As a newborn he was restless and had feeding problems. He learned to walk at 15 months of age, but his walking was unsteady until 2 years of age. At 4 years and 7 months of age he was brought to child neurologists due to delayed speech development and clumsiness. He was anxious and shy, and had tremor of the hands. In EEG irritation was detected. He had enuresis nocturna until 4 years of age. At 5 years of age his IQ was 70 (TML). At 7 years of age his development was estimated to be at 4.5 years of age. Due to moderate ID, he obtained special education. Further clinical studied revealed a normal brain CT, karyotype and urine metabolic screen at 11 years of age. A *de novo* variant in c.1904+3A>G predicted to impact splicing in *UBA7* was identified. Previously, a homozygous variant p.(E397*) in *UBA7* was reported in a Pakistani family suggesting autosomal recessive inheritance. It has been speculated that this variant is a risk factor for milder cognitive disability and is present in higher frequencies in South Asian population (Harripaul et al. 2018).

**FIN46-3** is a 50-year-old male, and the II/2 child of the family. He was born after an uncomplicated pregnancy and delivery. His birth weight was 3390 g, and had Apgar scores of 9/10. The first symptom of disease was hypsarrhythmia starting at 3 months of age followed by delayed psychomotor development. He learned to speak some words and learned to walk at 3 years of age, although his walking was atactic. He never learned to eat by himself. At 7 years of age his level in the Vineland test was 1 year and 3 months. From 7 years of age onwards he has been non-ambulatory. He has profound ID. He has large ears (length 8 cm) (Suppl Fig. 1c). He is sensitive to touch, has hypnic jerks, and screams at nights in addition to scoliosis. His feet are small (length 21 cm). A heterozygous *de novo* variant p.(R377S) in *DDX47* (OMIM # 615428) was identified.

**FIN-ID9-3** is a II/3 male child born to consanguineous parents after a normal pregnancy and delivery. His birth weight was 3180g, height 49 cm, head circumference 35,5 cm, and Apgar scores of 8/9/8. As a newborn, he presented with prominent forehead, exophthalmos and a flat nose (Figure S1C). Pes equinovarus and hernia inguinalis were operated on at one month of age. Soon after the birth he suffered from poor eating and swallowing, and a gastrostoma was set at 13 months of age. At 5 months of age he started to get absence seizures. He has been hypotonic since birth. He cannot speak and has severe psychomotor developmental delay. After repeated respiratory infections asthma was diagnosed 4 years of age. He was prone to vomit easily. He learned to walk at 5.5 years of age. He got encephalitis at 7 years of age but completely recovered. He has been extensively studied for metabolic diseases. His liver and muscle biopsies were normal. His pubertal development is normal. His hearing and vision are normal. Karyotype, molecular karyotype, urine metabolic screen, EEG, and brain MRI were normal. At 13 years of age, hip luxation and pain led to a loss of walking. He has short stature (-3 SD). He is currently 27 years old and lives in a home for handicapped individuals. A *de novo* variant p.(S637N) in *DHX58* (OMIM # 608588) was identified.

*Candidate variants in potential novel genes*

**FIN4-3** is a 26-year-old male, and the II/2 child. He was born at 40+2 weeks of pregnancy. His birth weight was 4065 g, and length 53 cm. He was diagnosed with Lennox-Gastaut epilepsy at 7 months of age followed by developmental delay, moderate to severe ID, and unclear speech. He went to a special school. His behavior was characterized by aggressive bursts and restlessness. His brain MRI was normal. His metabolic screen was normal. He has a pollen allergy. A mixture of two variants at the same position p.(Y757*) + p.(Y757=) was identified in *NTRK1.* The phenotype is not compatible to those due to *NTRK1* variants, which cause insensitivity to pain and anhidrosis (OMIM # 191315). Thus, the significance of the variants remains unknown.

**FIN21-3** is a 31-year-old male, and the I/3 child of the family. His birth weighed was 3930g, height 52 cm, head circumference of 37 cm, and Apgar scores of 8/8/9. He learned to walk at 19 months of age. His speech development was normal. At 3 years and 5 months of age he was evaluated because of slow growth. His bone age was delayed -3 SD. He has personal facial features, metopic suture (Fig 1b), high forehead, high palate, large eyes, helix asymmetry, short and broad hands, and mild ID (IQ 63). His inguinal hernia was operated on at 4 years of age. At 9 years of age his bone age was delayed -4.5SD. His growth hormone level and thyroid functions were normal. He did not have coeliac disease but did have obstipation. Karyotype and urine metabolic screen were normal. In EEG focal irritation was detected, but he did not have epilepsy. His brain MRI displayed enlarged ventricles. He studied in a small group in a normal school. In adulthood his height is 169 cm (-2SD), his BMI 37, and his head circumference is 61,5 cm. His pubertal development has been normal. Despite his young age, he has had repeated lung embolisms. He has had migraine attacks, and a therapy resistant panic disorder for several years. His parents are second cousins. A homozygous variant in exon 3 of the *SYPL1* gene, p.(C51Y) was identified (OMIM # 616665). When screening 263 individuals for the variant seven carriers were found in North-Eastern Finland showing a carrier frequency of 1:37.

**FIN23-3** is a 20 years old man whose birth weight was 2595g, height 49.5 cm, head circumference 33 cm. He was diagnosed with a rotator nystagmus at three months of age followed by developmental delay. He has progressive cerebral atrophy, cerebral ataxia, ID (at the border of mild to moderate), congenital body hypotonia, reduced visual acuity due to illness of visual cortex in cerebrum, central scotoma, strong myopia, mild sensorineural deafness, and white matter disease in the brain. In brain MRI delayed myelination, defective white matter signaling, corpus callosum hypoplasia, progressive atrophy of cerebellum has been detected (Suppl Fig. 3C). At 20 years of age his height is 158 cm and weight 48.5 kg. Localized epilepsy started at nine years of age. He spoke his first words at ~5 years of age. He uses signs to support communication. The patient uses a walking stand because of his cerebral ataxia. He also has fine motor problems and his hands suffer from athetosis. Molecular karyotype was normal. We identified a *de novo* missense variant p.(K181E) in the *ITPR2* gene (OMIM # 600144).

**FIN27-3** is a 17-year-old male, and the only child of his parents. He was born at 41+3 weeks of pregnancy. His birth weight was 3200g, height 50 cm, and head circumference 34.8 cm. As a newborn he had respiratory stridor. Since, 8 months of age he has been studied due to psychomotor developmental delay. He learned to walk at 3.5 years of age. He had muscle hypotonia. He did not learn to speak. He has obtained physiotherapy and speech therapy. He has syndromic facial features (Fig. 1c) and severe ID. The distal phalanges are short in his hands. At 14 months of age his brain MRI showed thin corpus callosum, abnormal lateral ventricles and gyri, reduced white matter volume, and normal myelination. Frontally cortical liqvor spaces and fissura sylviae were enlarged. At 9 years of age he had a febrile seizure. The EEG displayed a generalized disturbance without epileptiform activity. Hearing is normal. He shakes his head and upper legs when happy. All laboratory investigations including karyotype, FMR1 gene test, urine metabolic screen and lactate were normal. His adult height is 169 cm and head circumference 56.5 cm. Based on the clinical findings he has a syndromic phenotype. A *de novo* missense variant p.(F434L) was identified in *ZKSCAN1* (OMIM *#* 601260)*.*

**FIN32-3** is a 35-year-old female, and the I/3 child of the family. Her birth weight was 3880g, and Apgar scores 10/10. Her early development was normal. She was evaluated at 5 years of age due to slender habitus (height – 1.8 SD) and psychomotor delay. Mild dysphasia was detected and she received speech therapy. Her neurological status was normal. She had mild ID, her personality is shy and she has aggressive bursts. EEG was normal. She went to a special school and is now working in childcare as a helper. A *de novo* missense variant p.(D889E) in *ZFR* (OMIM # 615635) was identified.

**FIN45-3** is a 51-year-old female, and the III/4 child of the family. Her mother had a normal pregnancy and her delivery was uneventful. She presented with hypsarrhythmia and infantile spasms at 6 months of age. She never learned to speak or walk. She has profound ID. As an adult, grand mal type epileptic seizures occur 1-2 times per month with high pitched shouts. Saliva production is strong with saliva spills during eating. Her adult height is 161 cm, head circumference 54 cm. She has small feet (length 19.5 cm). Blood values are normal except low albumin 33g/l (36-45 g/l). A *de novo* splice site variant c.294-2A>G in RNA Polymerase II Subunit F *(POLR2F)* (OMIM # 604414).

**FIN47-2** is a 55-year-old male who has profound ID. He was born at breech presentation and had an Apgar score of 7. As an infant he was evaluated at the Children’s hospital in Helsinki due congenital hydrocephalus and diplegia spastica. He learned to walk at 7 years of age but lost the skill as an adult. He cannot speak or eat by himself. Otherwise the data is scarce regarding his hospital records. His head circumference is 59 cm. His EEG is normal. At 52 years of age his brain CT showed mild cerebral atrophy. He has nystagmus and dysmorphic ear lobes but no facial dysmorphism. A heterozygous variant p.(I3989V) in *DNAH3* (OMIM # 603334) was identified in WES which was absent in the healthy sibling. Parental samples were unavailable.

**FIN-ID3: FIN-ID3-1** is a 68-year-old female, and the I/5 child of the family. Her birth weight was 3300g, and her height 48 cm. Her early development was normal. Her adult height is 147 cm and head circumference is 55.3 cm. She has some dysmorphism in her face, such as hypertelorism and thin upper lip. Her neurological status is normal. She could not complete normal school but can read and write. She has mild ID and her IQ is 52. She was diagnosed with Type II diabetes at 33 years of age, which is now leading to blindness due to diabetic retinopathy. Her karyotype is a normal 46, XX. Her personality is peaceful and shy. She has never worked independently. She lives with a family where she gets help with everyday tasks.

**FIN-ID3-3** is a 56-year-old male, the IV/5 child of the family, and the younger brother of FIN-ID3-3. His birth weight was 3900 g, and his height 50 cm. He was operated on due to a cleft lip at 6 months of age. He went to a special school and could not complete compulsory army service. His adult height is 163 cm and his head circumference is 56 cm. He has strabismus, hypertelorism, and thin upper lip. His personality peaceful and shy. His karyotype is 46, XY. He has never worked independently. He lives independently in his childhood home. The siblings have a novel homozygous splice variant c.717+1G>A in *ERGIC3* (OMIM # 616971)*.* Their unaffected brother does not carry the variant whereas their youngest sister, who is healthy, is also a carrier of the variant.

**FIN-AIC2-3** is a 31 years old female, who is an only child. Pregnancy and delivery were normal. Her birth weight was 4150 g and height 54 cm. She was studied due infantile spasms at 3 months of age. She had severe hypotonia. In the EEG hypsarrhythmia was detected. The ophthalmological investigation found depigmented white spots in retina and coloboma in the left eye. Brain CT showed hypoplasia of corpus callosum. Metabolic screen, karyotype, and molecular karyotype were normal. As an adult she has severe neurodevelopmental delay. She needs constant help. We identified compound heterozygous variants in *KIF1B* [(p.(P689L); p.(P848L)] (OMIM # 605995).

*Structural variants*

**FIN10-3** is a 23-year-old female, and the II/2 child of the family. Her birth weight was 2440g, and height was 45 cm. She was small for her date of birth, since she was not premature. She was diagnosed with alloimmunothrombocytopenia as a newborn. She has severe microcephaly (-5 SD), unique facial features (Fig.1d), micrognathia, and a dysmorphic right ear. Her epilepsy started before one year of age. She has profound psychomotor developmental delays and growth delays. Her adult height is 144 cm (-4SD). She does not speak, has autistic features, and cannot eat or drink by herself, thus a gastrostoma was inserted. Her gait is unsteady, and she has stiffness. The EEG displayed focal irritation, and unspecific epileptic seizures have been detected. Her karyotype, brain MRI, and metabolic screen were normal.

Since no putatively causal variants were identified through the study of her and the parents’ WES data, molecular karyotyping was performed. The patient was shown to carry a pathogenic mosaic interstitial deletion of 2.7 Mb extending from cytogenetic band 21q11.12 to 21q22.2 (chr21:37652840-40383486, hg19) present in 22% of cells.

The molecular karyotype result is marked as:

arr[GRCh37]21q22.12q22.2(37652840_40383486)x1[0.22]pat/ 21q22.11q22.3(34343695_48098824)x2 hmz mat[0.78]/ 21q21.3q22.11(27178550_34292163)x2 hmz mat[0.3]/21q21.1q21.3(17531989_27129093)x2 hmz mat [0.15].

The FISH results are marked as:

Uncultured interphases:

mos nuc ish del(21q22.13)(RP11-777J19-)[47]/21q22.13(RP11-777J19x2)[153]

PHA-cultured cells:

mos nuc ish del(21q22.13)(RP11-777J19-)[69]/21q22.13(RP11-777J19x2)[231] (interphases)

mos ish del(21q22.13)(RP11-777J19-)[6]/21q22.13(RP11-777J19x2)[19] (metaphases)

In addition to *DYRK1A* deletion mosaicism, complex mosaicism of three different homozygosity regions, 21q22.11q22.3 (78%), 21q21.3q22.11 (30 %) and 21q21.1q21.3 (15 %), were detected most probably presenting uniparental disomy (UPD). SNP-genotype analysis from FIN10 and her parents showed that deletion was originated in paternal chromosome 21 and that uniparental disomies were maternal in origin. The deletion mosaicism was confirmed by fluorescent in situ hybridization (FISH) in which the deletion cell line was detected in 23.5% of uncultured and 23 % of cultured interphases and 24 % of metaphases.

A 21q22.12q22.2 deletion region involving fifteen protein coding genes of which five have a known association with specific clinical disorders. Of these, a deletion of the *DYRK1A* gene (OMIM #600855, Dual-specificity tyrosine phosphorylation-regulated kinase 1A) is associated with microcephaly, ID, speech impairment, motor difficulties, feeding problems, short stature, and distinct facial features (#614104, Mental retardation autosomal dominant 7). The finding is in agreement with the clinical features of FIN10-3.

**FIN43-3** is the first child of the family. He was born at 40+3 weeks of pregnancy with a birth weight of 3030g (-1.5SD), a height of 47 cm (-2.3 SD), and a head circumference of 29.5cm (-4.6 SD). Apgars were 9/10/10. He walks with aids, speaks singleton words, and is social. At 3 years his height was 85 cm (- 3.5 SD), weight was 10.8 kg and head circumference was 39.2 cm (- 9,6 SD). In his brain MRI as newborn and at 17 months of age, microcephaly, simplified gyral pattern, and partial corpus callosum agenesis were detected. During the mother´s second pregnancy, delayed growth of the head was noticed at 18+3weeks. In neuropathological studies microcephaly was detected. In molecular genetic studies the healthy father, the first child and the fetus had a heterozygous 1,25 Mb interstitial microdeletion at 16p13.11. The deleted region contains 11 genes, of them only *NDE1* has been associated with disease (Figure S2B).

**FIN48-2** is a 62-year-old male, and the II/6 child of the family. His birth weight was 2900g and his height was 49cm. His early phases were normal. During childhood he was evaluated due to a delay in speech development. He has spoken simple words with repetition, but has lost his speech some years ago. He has severe ID (IQ 40), autistic features, high pain tolerance, and spasticity. He has difficulties in walking and is sitting in a wheelchair. With age his behavior has become restless. He has laughing bursts, aggressions, and self-mutilation. He has athetotic movements in his upper arms, obstipation, and urine incontinence. A deletion at 22q13.3 encompassing *SHANK3* was identified in CNV analysis (Figure S2C). The phenotype is compatible with Phelan-McDermid syndrome (Chromosome 22q13.3 deletion syndrome) (OMIM # 606232). The deletion was absent in the mother. The paternal sample was unavailable.

**Supplemental Figure legends**

**Figure S1A.** Individuals with known pathogenic variants identified in this study. FIN3-3 with a variant p.(E198K) in *PPP2R5D*, FIN11-3 with a variant p.(E665K) in *CYFIP2* (A-B), FIN24-3 with a variant p.(S348L) in *ACTB* (A-B), and FIN52-2 with a *de novo* variant p.(R3070C) in *HUWE1.*

**Figure S1B.**  Individuals with novel pathogenic/likely pathogenic variants identified in this study. FIN6-3 with p.(D432fs) in *HNRNPK* (A-D), FIN14-3 with p.(F53del) in *CTPB1 (A-B)*, FIN20-3 with p.(K620*) in *CHAMP1* (A-B), FIN23-3 with de novo variant p.(K181E) in *ITPR2,* FIN33-3 with p.(R1198S) in *RAI1,* FIN36-3 with compound heterozygote variants p.(E1668fs) + (c.2315-28A>G) in *LAMB1,* FIN42-3 and FIN42-7 with p.(P413L) in *P4HTM*, FIN49-1 with a *de novo* variant p.(M631V) in *SCN1A,* FIN53-1 with a heterozygous variant p.(T1303I) in *TRIO* and p.(Q318P) in *SON,* FIN-ID4-3 with a variant p.(Q1082*) in *KIAA2022,* and FIN-ID10-3 with a *de novo* p.(A2265fs) variant in *ANKRD11.*

**Figure S1C.** Individuals with a phenotypic expansion in a known gene identified in this study. FIN5-3 with p.(I908V) in *SAMD9L* (A-D), FIN8-3 with p.(T326K) in *BCL11A*, FIN28-3 with p.(V392fs) in *MECP2,* ~~and~~ FIN35-3 with p.(E264Q) in *MYT1* and FIN-AIC3 -3 with homozygous splice variant c.1177+9T>C in *ZC3H14.*

**Figure S1D.** Individuals with alternate inheritance model in known genes. FIN-ID9-3 with a *de novo* variant p.(S637N) in *DHX58.*

**Figure S1E.** Individuals with candidate variants in potential novel genes. FIN21-3 with a novel autosomal recessive variant p.(C51Y) in *SYPL1* (A-E), FIN27-3 with p.(F434L) in *ZKSCAN1,* FIN-ID3-1 (A-C) and FIN-ID3-3 (A-C) with a novel autosomal recessive splice site variant (c.717+1G>A) in *ERGIC3* and FIN45-3 with a novel autosomal recessive splice site variant (c.294-2A>G) in *POLR2F.*

**Figure S2.** A. MRI of FIN36-3 with *LAMB1* variants. T-sagittal image. a) shows hypoplastic corpus callosum (thin arrow). Brain stem and vermis (arrow) are hypoplastic. T2 axial image (b) shows mild hypoplasia of cerebellar hemispheres with small cystic lesions (arrows). T2 axial image (c) shows dilated lateral ventricles and symmetric white matter abnormality. There is cobblestone malformation (thin arrow) with posterior-anterior gradient. The layer of ectopic cortex is lining lateral ventricles (arrowhead). In flair image (d) dentate nuclei are hyperintense (arrows). Optic nerves were atrophic (not shown).

B. MRI T2 axial image of FIN38-3 with *CRADD* variants. (a) showed thick cortex with broad gyri compatible with pachygyria. Coronal flair image (b) shows few unspecific punctate white matter lesions (arrows).

C. T2 weighted MRI images of FIN23-3 with *de novo* *ITPR2* variant at 10 years of age. a) show cerebral and cerebellar white matter hypomyelination. b) shows cerebellar atrophy. c) shows Vermian atrophy and thin corpus callosum.

**Figure S3.** Chromosomal and molecular karyotype findings in this study. Only one red signal covering the *DYRK1A* gene (RP11-777J19) was detected in 24% of metaphases whereas two green signals of the 21q21.1-control region (RP11-687D14) was seen in all cells found in FIN10-3 in FISH (A), SNP microarray analysis showing a complex chromosome 21 uniparental disomy mosaicism with a mosaic interstitial 21q22.12q22.2 deletion covering DYRK1A gene (area between red lines) in FIN10-3 (B), Heterozygous deletion at 16p13.11 in FIN43-3 (C), and Heterozygous 22q13.3del covering *SHANK3* in FIN48-3 detected via exome sequencing (D).

**Figure S4:** Potential mechanisms for the partial rescue of the 21q22.12q22 (*DYRK1A*) deletion by development of mitotic distal 21q iUPDs. A) A) Independent formation of three different segmental UPDs. B) Formation of segmental iupd of 21q22.11q22.3 region (48%) followed by formation of iupd(22q21.3q22.3) (15%) and iupd(21q21.1q22.3) (15%), respectively. P=paternal chromosome, M=maternal chromosome.

***References***

Bainbridge MN, Wiszniewski W, Murdock DR, et al (2011) Whole-genome sequencing for optimized patient management. Sci Transl Med 3:87re3. https://doi.org/10.1126/scitranslmed.3002243

Bebbington A, Percy A, Christodoulou J, et al (2010) Updating the profile of C-terminal MECP2 deletions in Rett syndrome. J Med Genet 47:242–248. https://doi.org/10.1136/jmg.2009.072553

Challis D, Yu J, Evani US, et al (2012) An integrative variant analysis suite for whole exome next-generation sequencing data. BMC Bioinformatics 13:8. https://doi.org/10.1186/1471-2105-13-8

Chang CC, Chow CC, Tellier LC, et al (2015) Second-generation PLINK: rising to the challenge of larger and richer datasets. Gigascience 4:. https://doi.org/10.1186/s13742-015-0047-8

Fromer M, Purcell SM (2014) Using XHMM software to detect copy number variation in whole-exome sequencing data. Curr Protoc Hum Genet 81:7.23.1-7.23.21. https://doi.org/10.1002/0471142905.hg0723s81

Gambin T, Akdemir ZC, Yuan B, et al (2017) Homozygous and hemizygous CNV detection from exome sequencing data in a Mendelian disease cohort. Nucleic Acids Res 45:1633–1648. https://doi.org/10.1093/nar/gkw1237

Harripaul R, Vasli N, Mikhailov A, et al (2018) Mapping autosomal recessive intellectual disability: combined microarray and exome sequencing identifies 26 novel candidate genes in 192 consanguineous families. Mol Psychiatry 23:973–984. https://doi.org/10.1038/mp.2017.60

Kroepfl T, Petek E, Schwarzbraun T, et al (2008) Mental retardation in a girl with a subtelomeric deletion on chromosome 20q and complete deletion of the myelin transcription factor 1 gene (MYT1). Clinical Genetics 73:492–495. https://doi.org/10.1111/j.1399-0004.2008.00982.x

Krumm N, Sudmant PH, Ko A, et al (2012) Copy number variation detection and genotyping from exome sequence data. Genome Res 22:1525–1532. https://doi.org/10.1101/gr.138115.112

Li H, Durbin R (2009) Fast and accurate short read alignment with Burrows-Wheeler transform. Bioinformatics 25:1754–1760. https://doi.org/10.1093/bioinformatics/btp324

Yang H, Wang K (2015) Genomic variant annotation and prioritization with ANNOVAR and wANNOVAR. Nat Protoc 10:1556–1566. https://doi.org/10.1038/nprot.2015.105
