# Supplementary figures and images for: Exome sequencing reveals predominantly de novo variants in disorders with intellectual disability (ID) in the founder population of Finland
Source: Hum Genet. 2021 Mar 12;140(7):1011–29. doi: 10.1007/s00439-021-02268-1 (PMC8197721; doi:10.1007/s00439-021-02268-1)

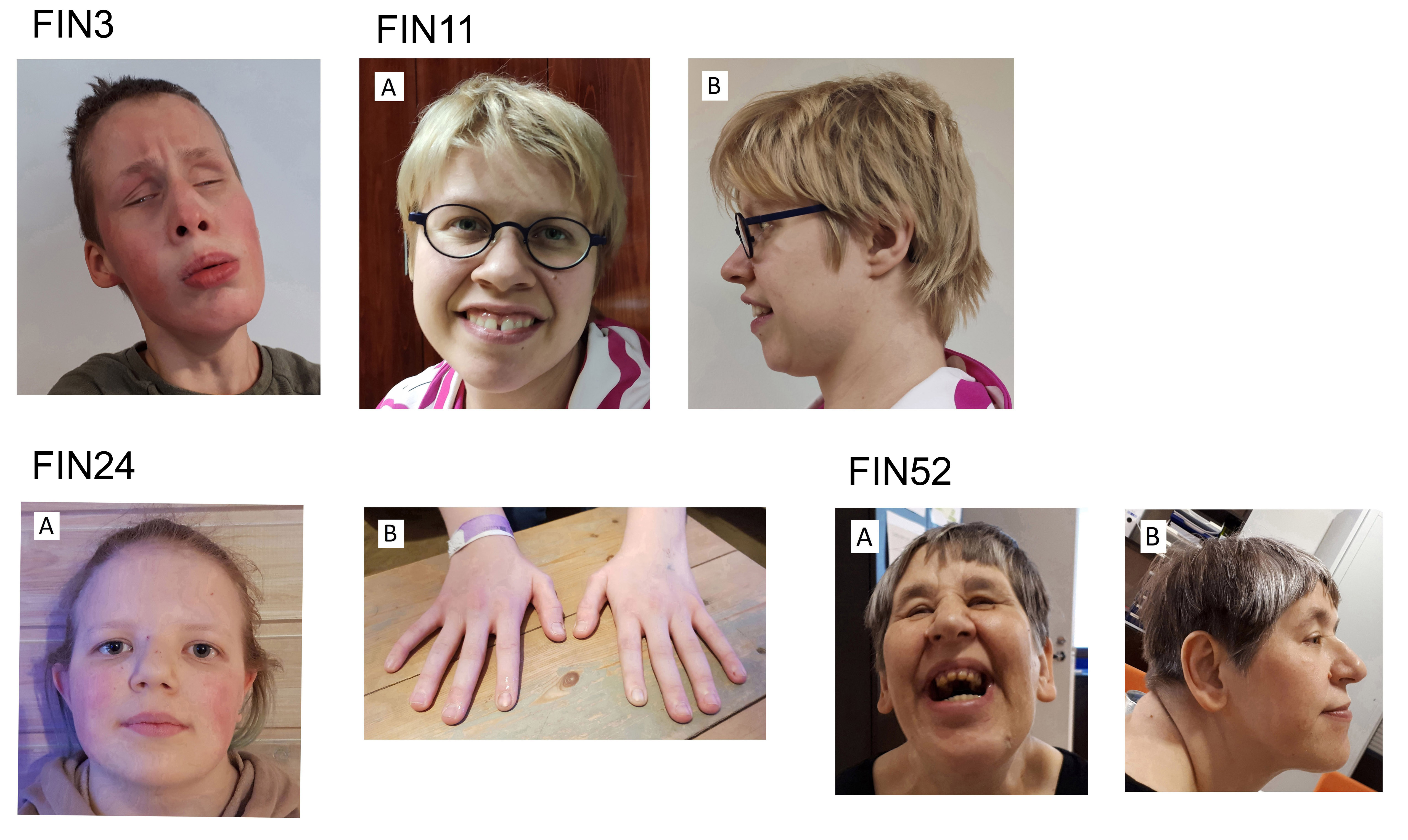

Supplement: Supplementary file 2 — Supplementary file2 (JPG 1363 KB) [file 439_2021_2268_MOESM2_ESM.jpg]

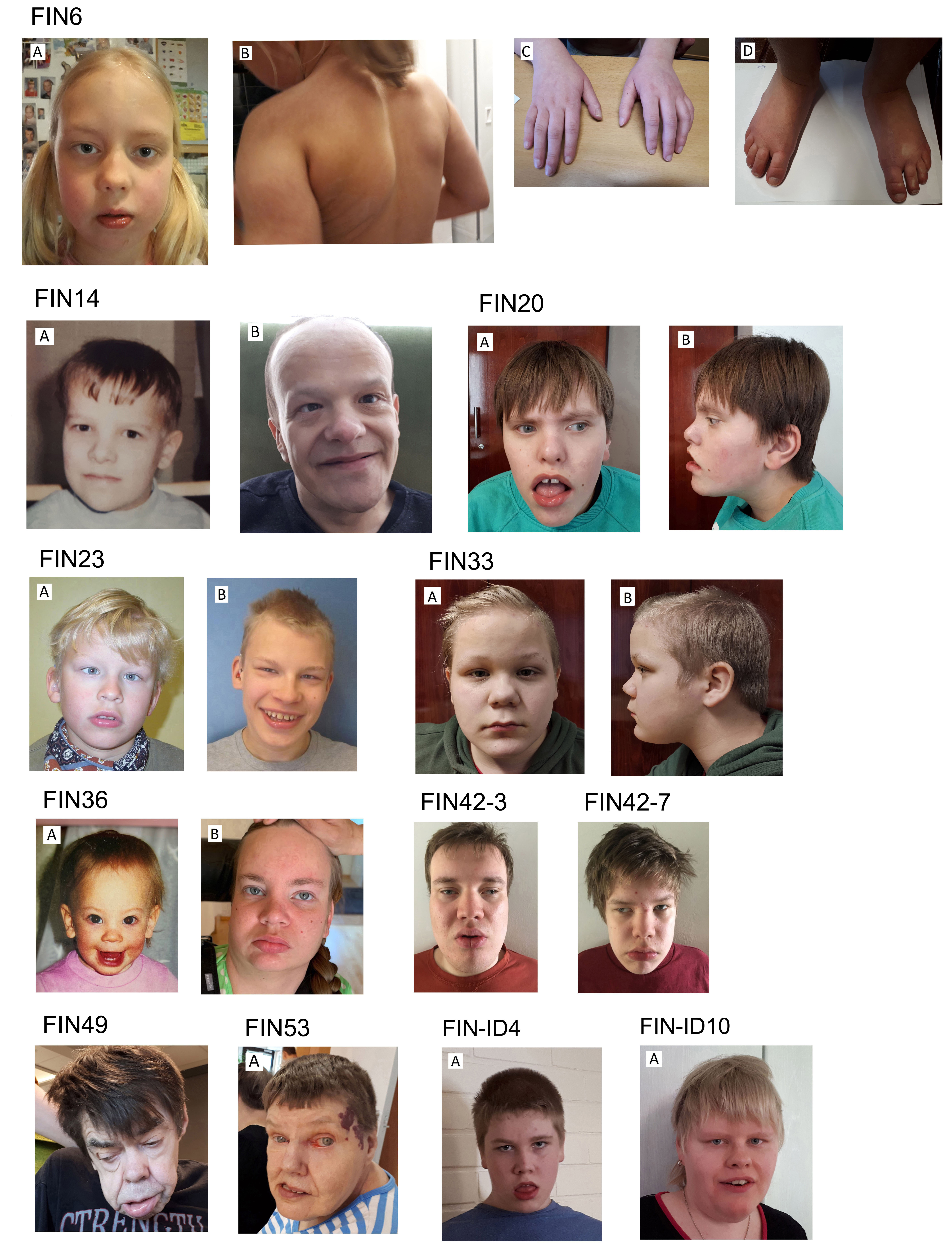

Supplement: Supplementary file 3 — Supplementary file3 (JPG 2865 KB) [file 439_2021_2268_MOESM3_ESM.jpg]

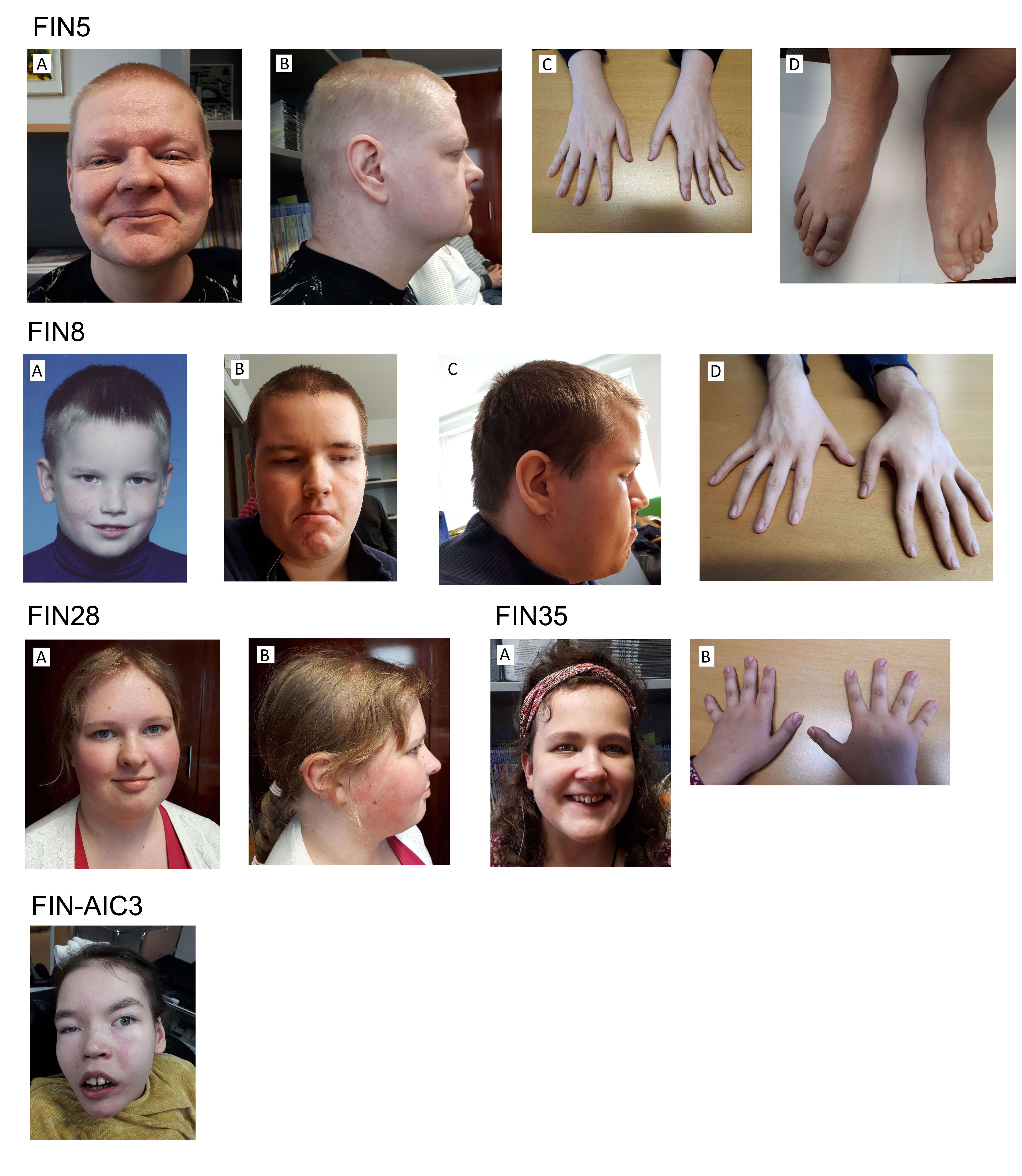

Supplement: Supplementary file 4 — Supplementary file4 (JPG 2293 KB) [file 439_2021_2268_MOESM4_ESM.jpg]

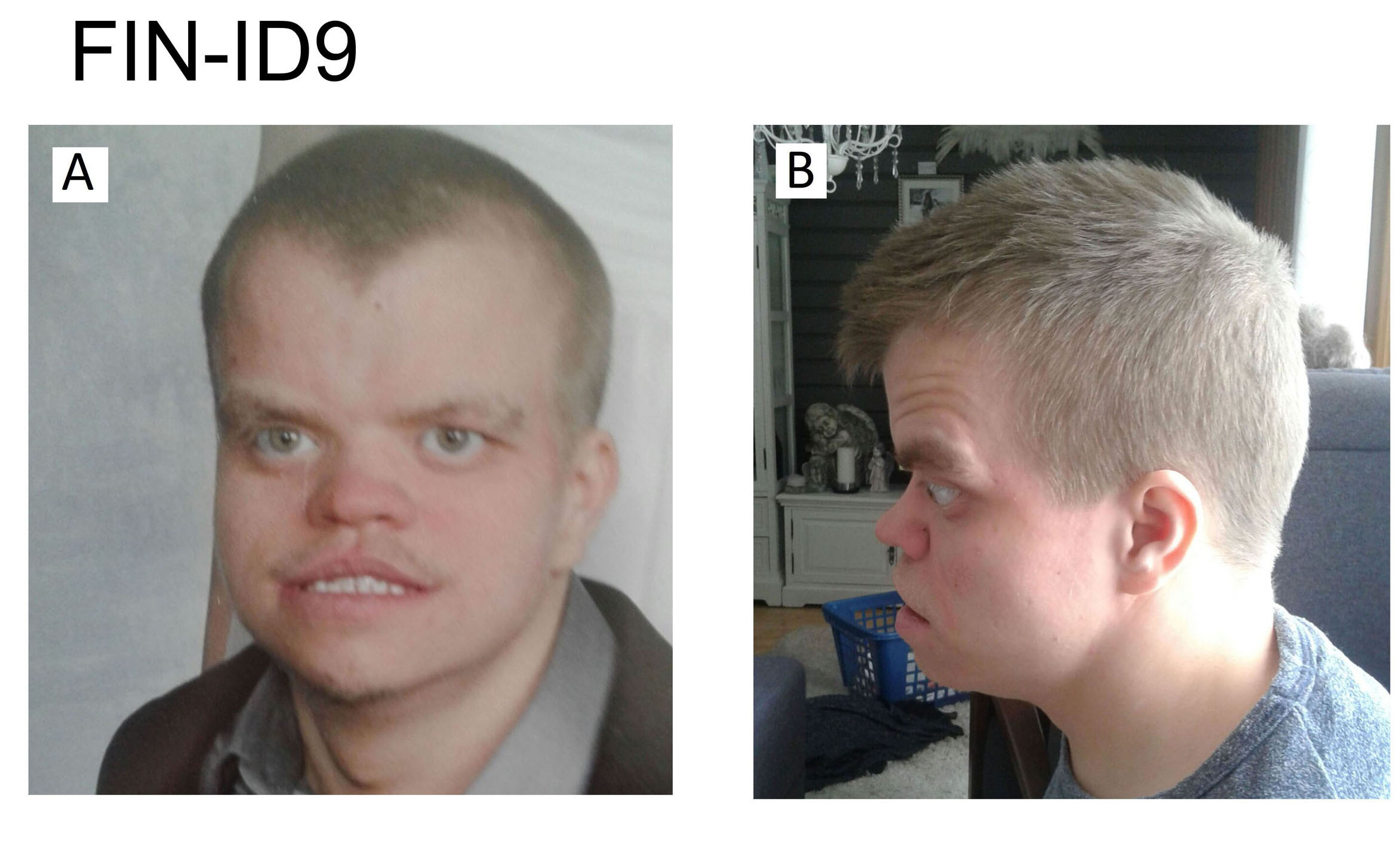

Supplement: Supplementary file 5 — Supplementary file5 (JPG 297 KB) [file 439_2021_2268_MOESM5_ESM.jpg]

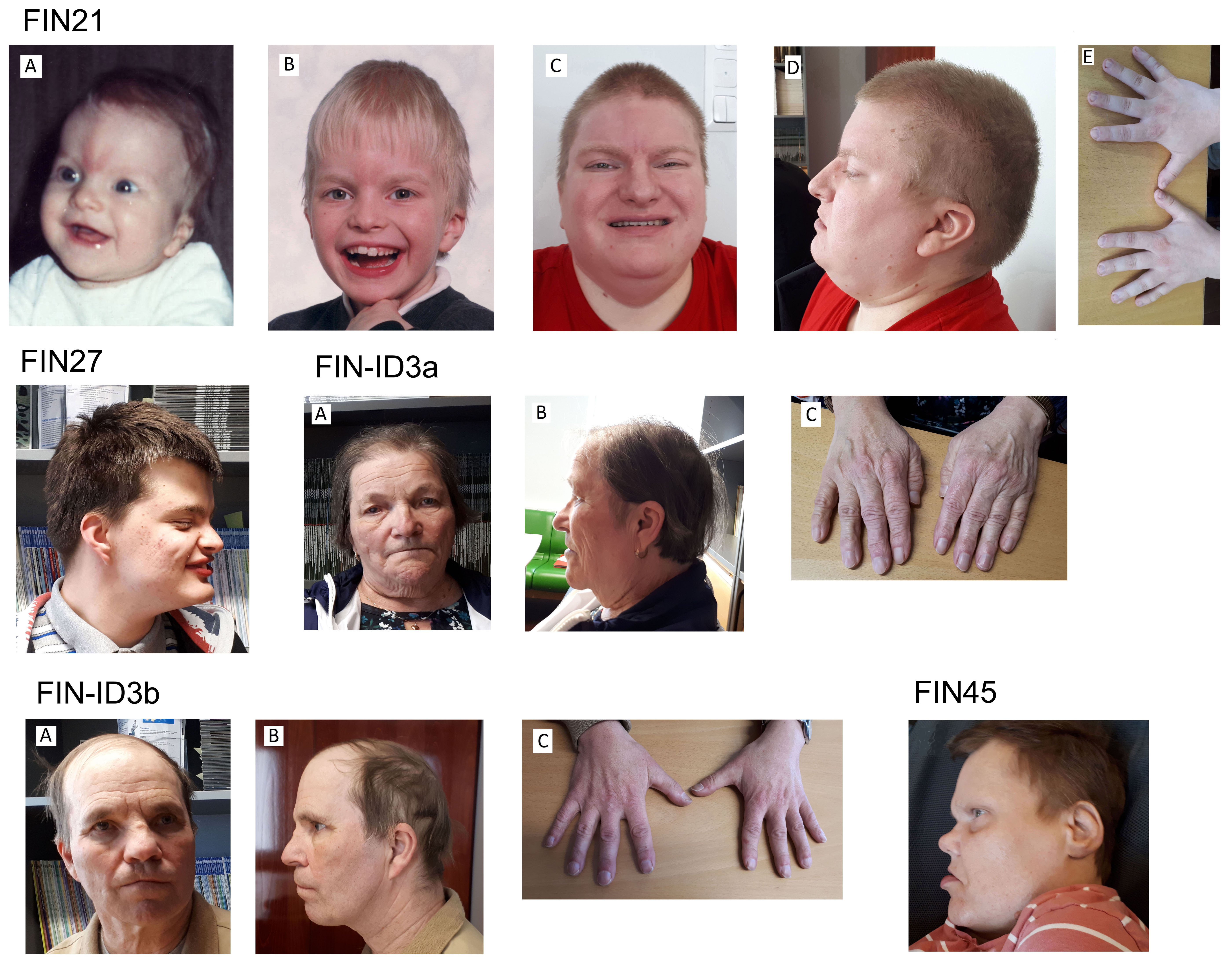

Supplement: Supplementary file 6 — Supplementary file6 (JPG 2019 KB) [file 439_2021_2268_MOESM6_ESM.jpg]

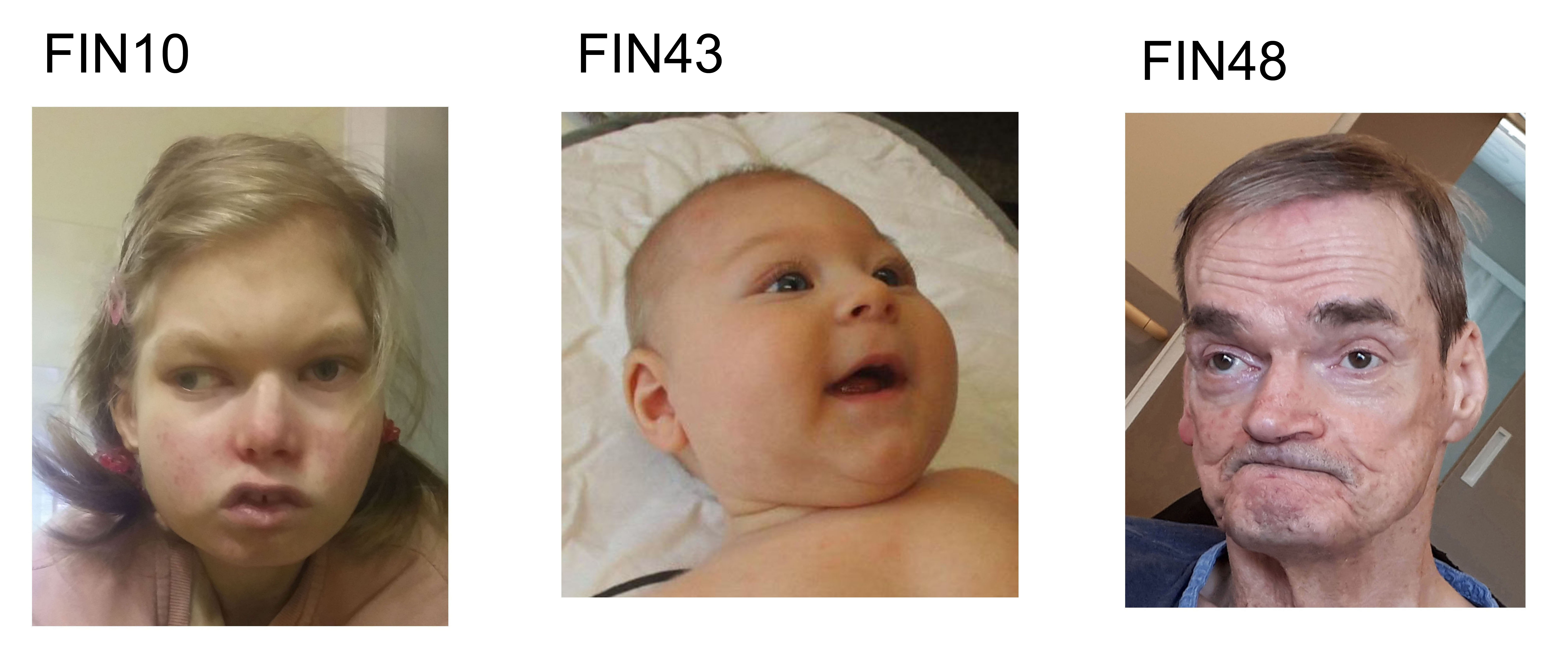

Supplement: Supplementary file 7 — Supplementary file7 (JPG 1244 KB) [file 439_2021_2268_MOESM7_ESM.jpg]

## Slide 1
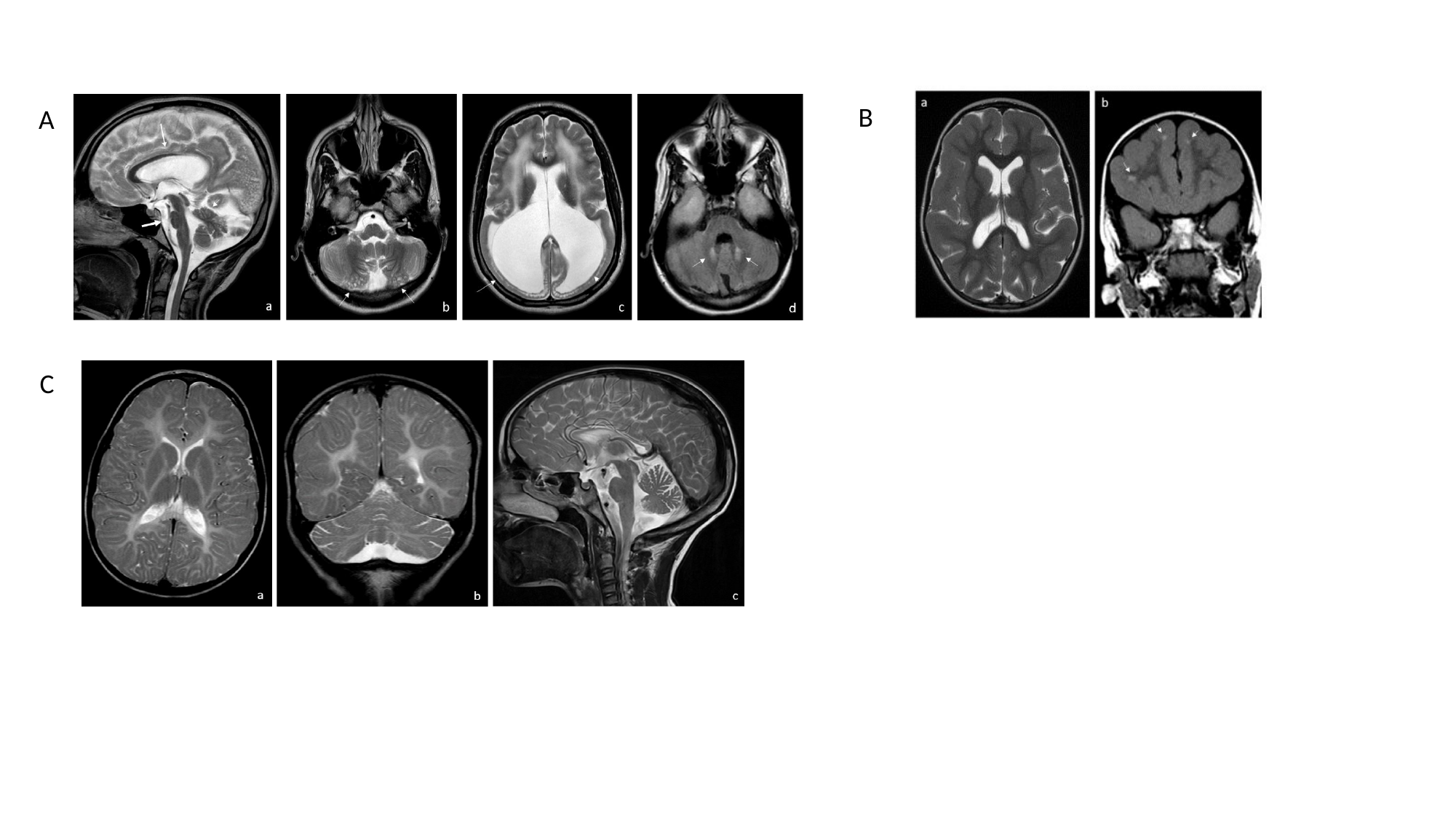

B
A
C

Supplement: Supplementary file 8 — Supplementary file8 (PPTX 2534 KB) [file 439_2021_2268_MOESM8_ESM.pptx]

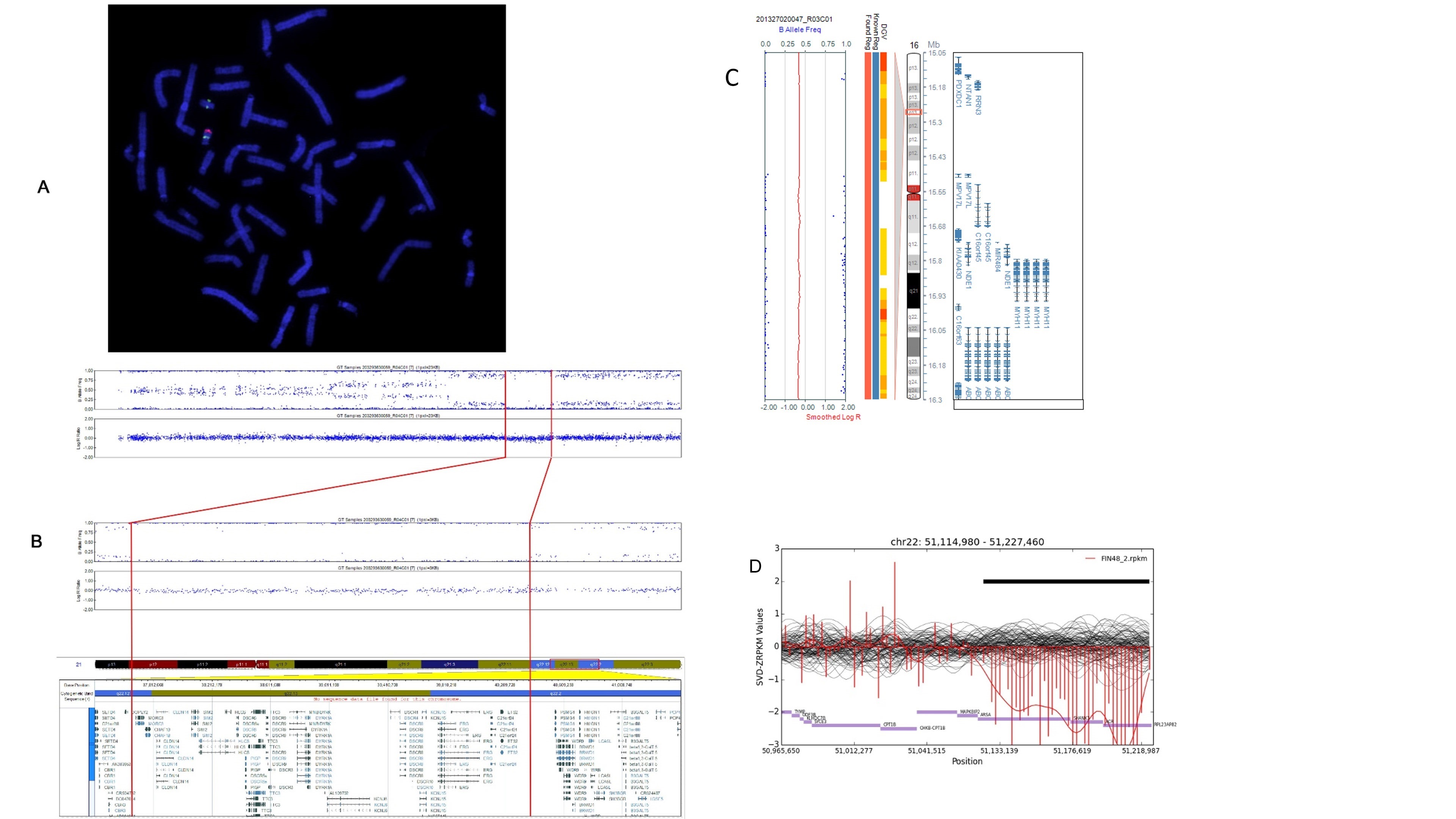

Supplement: Supplementary file 9 — Supplementary file9 (JPG 853 KB) [file 439_2021_2268_MOESM9_ESM.jpg]

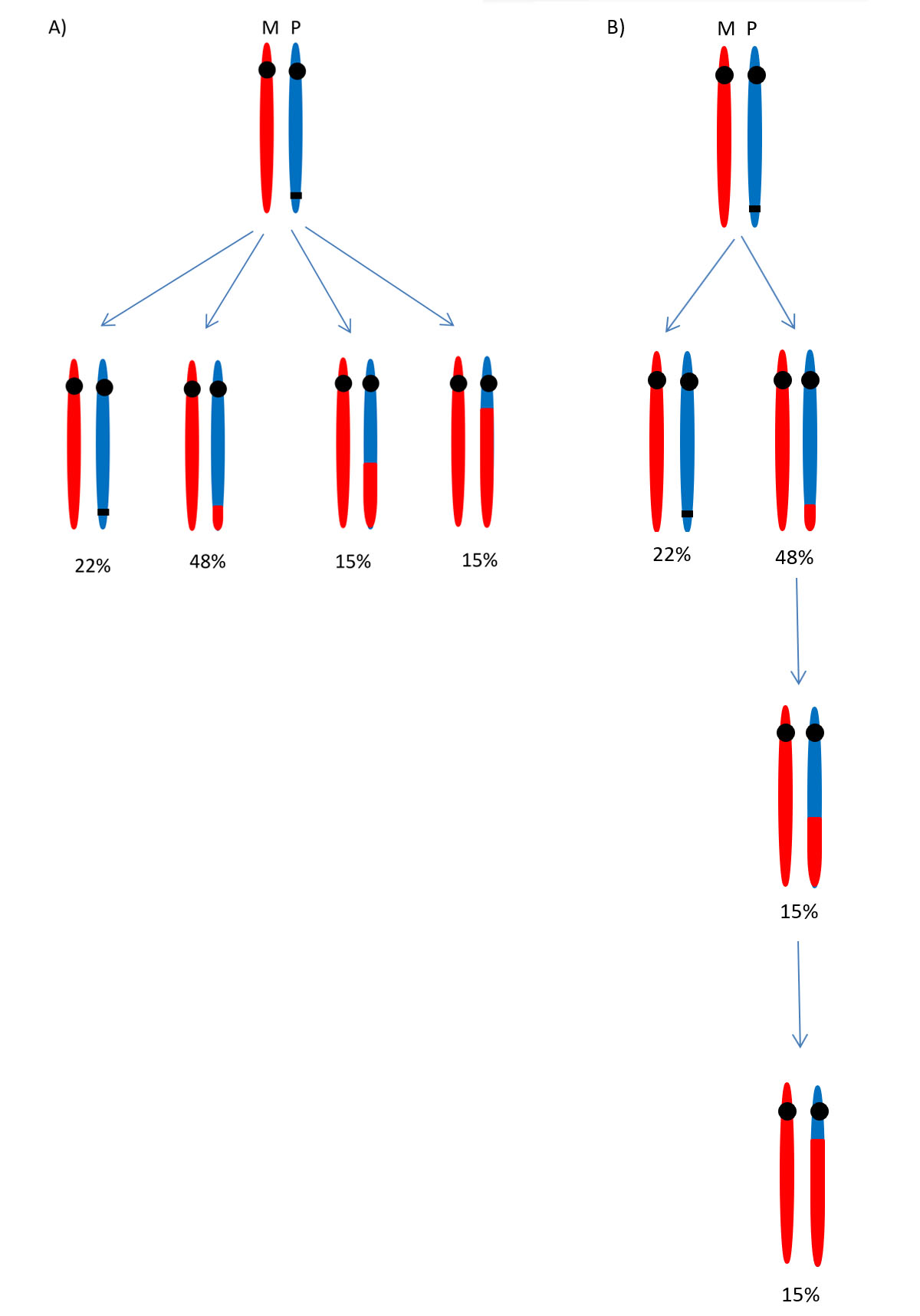

Supplement: Supplementary file 10 — Supplementary file10 (JPG 140 KB) [file 439_2021_2268_MOESM10_ESM.jpg]
